# A Comprehensive Review of Deep-Learning-Based Methods for Image Forensics

**DOI:** 10.3390/jimaging7040069

**Published:** 2021-04-03

**Authors:** Ivan Castillo Camacho, Kai Wang

**Affiliations:** GIPSA-Lab, Grenoble INP, CNRS, Université Grenoble Alpes, 38000 Grenoble, France; ivan.castillo-camacho@gipsa-lab.grenoble-inp.fr

**Keywords:** image forensics, fake image detection, deep learning, neural network, Deepfake

## Abstract

Seeing is not believing anymore. Different techniques have brought to our fingertips the ability to modify an image. As the difficulty of using such techniques decreases, lowering the necessity of specialized knowledge has been the focus for companies who create and sell these tools. Furthermore, image forgeries are presently so realistic that it becomes difficult for the naked eye to differentiate between fake and real media. This can bring different problems, from misleading public opinion to the usage of doctored proof in court. For these reasons, it is important to have tools that can help us discern the truth. This paper presents a comprehensive literature review of the image forensics techniques with a special focus on deep-learning-based methods. In this review, we cover a broad range of image forensics problems including the detection of routine image manipulations, detection of intentional image falsifications, camera identification, classification of computer graphics images and detection of emerging Deepfake images. With this review it can be observed that even if image forgeries are becoming easy to create, there are several options to detect each kind of them. A review of different image databases and an overview of anti-forensic methods are also presented. Finally, we suggest some future working directions that the research community could consider to tackle in a more effective way the spread of doctored images.

## 1. Introduction

Given our era of advanced technology and the high availability of image-editing tools that make it extremely easy and fast to alter and create fake but realistic images, the trust of digital images has diminished. We can no longer easily accept an image as proof of an event without asking ourselves if the image has been modified. This has been in a continuous development together with the easy accessibility of tools used to create tampered-with content and with the deep-learning advancements which have led to an increase in the realism of fake images or videos [1].

During recent years, an evolution of disinformation has appeared to manipulate and disrupt public opinion. This disinformation comprises sophisticated campaigns aided by doctored images with the goal of influencing economic and societal events around the world. Different kinds of problems related to the usage of tampered-with images have appeared in different fields and will get worse as both digital cameras and software editing tools become more and more sophisticated.

**Figure 1:**
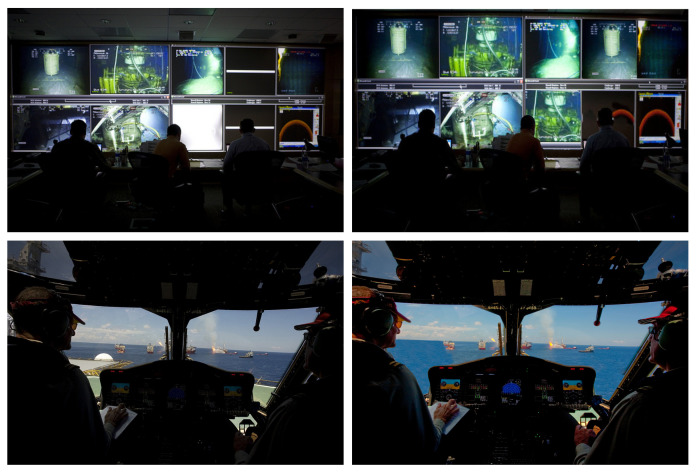
Examples of image forgery during the BP oil spill. First row shows how the original image was modified by copying some screens over the initially blank ones. On the second row, the helipad was removed in the tampered-with version. Images were obtained from the following webpage: https://www.cbsnews.com/news/bp-and-the-gulf-oil-spill-misadventures-in-photoshop/ accessed on 2 April 2021.

In July 2010, British Petroleum (BP) came under public outcry over several doctored images of its Gulf of Mexico oil spill response, as images were tampered with to indicate that BP staff were busier than they actually were. Figure 1 shows two pairs of the original (first column) and the tampered-with (second column) images. A spokesperson for the company eventually admitted that in one image (first row of Figure 1) two screens were actually blank in the original picture. On the second row of Figure 1, we see a photo taken inside a company helicopter which appeared to show it flying off the coast. It was later shown to be fake after Internet bloggers identified several problems, which suggested that the helicopter was not even flying. The problems included part of a control tower appearing in the top left of the picture, its pilot holding a pre-flight checklist, and the control gauges showing the helicopter’s door and ramp open and its parking brake engaged (Please refer to details presented at the following webpage: https://metro.co.uk/2010/07/22/bp-admits-to-doctoring-another-deepwater-horizon-oil-spill-image-456246/ accessed on 2 April 2021).

From this context, it is necessary to develop strategies and methods to allow the verification of the authenticity of digital images. Image forensics [2] is the science that can help us to know if the image was acquired by the claimed device or if the current state corresponds to the original captured image, with the objective of detecting and locating image forgeries. Image forensics techniques depend on the assumption that each stage of the image acquiring and processing, from the raw image to its compression, storage and post-processing, holds some inherent statistics and leaves a particular trace. It is then possible to infer the source of the image or decide whether it is authentic or tampered with by identifying the existence, lack or inconsistency of forensic traces that are inherent to the image itself.

The research on this field started around 20 years ago and has recently seen a boost with the latest deep-learning tools. The deep-learning framework [3] usually uses a hierarchical structure of artificial neural networks, which are built in a similar way to the neural structure of the human brain, with the neuron nodes connected to simulate a neural network. This architecture can approach data analysis in a non-linear way. The striking advantage of deep learning is its ability to automatically learn useful features from available data, allowing us to bypass the tedious procedure of handcrafted feature design. Regardless of the big progress in the computer vision field using deep-learning tools, the same strategies cannot be applied directly to the image forensics domain as the traces or fingerprints that we are looking for are normally not present in the visible domain. Most of the traces that we search are hardly perceptible by the human eyes. Therefore, as we can see later in this paper certain strategies have been proposed to cope with this difference.

Early surveys on image forensics [2,4,5,6,7] naturally focused mainly on conventional feature-based methods. Recent surveys [8,9] consider both conventional and deep-learning methods yet with a different focus or coverage from ours. For instance, ref. [8] mainly considers the detection of copy-move, splicing and inpainting, while we cover more image forensics problems including also the detection of routine image processing operations, the detection of synthetic images, etc.; Ref. [9] classifies existing methods from a machine learning perspective (e.g., supervised, unsupervised and anomaly detection) with a special and timely focus on Deepfake detection, while we classify with a rather comprehensive list of image forensics problems and focus on the particularities of deep network design for different problems. Other existing surveys have dedicated their reviews to presenting and analyzing the methods for one or several specific issues such as copy-move (and splicing) detection [10,11], computer-generated image detection [12], camera identification [13] and image source identification [14], while we attempt to have a broader coverage.

**Figure 2:**
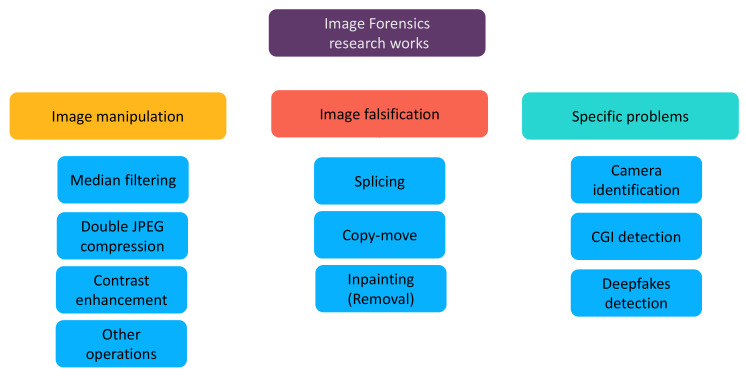
Classification diagram for deep-learning-based image forensics works. “CGI” means computer graphics image.

In this paper, we review existing deep-learning-based methods for a variety of image forensics problems. The research works presented in this survey are classified into three large groups: the detection of image *manipulations* (i.e., routine image processing operations such as median filtering and contrast enhancement), the detection of image *falsifications* which intentionally alter the semantic meaning of the image (e.g., copy-move, splicing and inpainting) and other specific forensic problems. We pay attention to special designs of the deep models and special features used on the network input. Considering the rapid advancement in the image forensics field and the difference between our review and existing ones as discussed in the last paragraph, we believe that our survey can be helpful for the research community and is complementary to previous reviews. Our classification of research works on image forensics is illustrated in Figure 2.

The remainder of this paper is organized as follows. We first present in Section 2 the datasets used for image forensics research which are vital for data-driven methods based on deep learning. Section 3, Section 4 and Section 5 are dedicated respectively to the presentation of deep-learning-based methods for the detection of routine image manipulations, the detection of intentional image falsifications and other specific forensic problems, in accordance with the classification mentioned above and shown in Figure 2. We present in Section 6 a brief review of anti-forensic methods which aim at defeating the analysis of forensic detectors. We conclude and suggest some future working directions in Section 7.

## 2. Datasets

Aside from the different models and different approaches, the access to a proper dataset is the first step and has a crucial role in the deep-learning paradigm to make it work properly. This means using a dataset that corresponds to the results a researcher wants to predict. The dataset should match the problem context including the acquiring and any processing steps. Constructing a dataset is a time-consuming task which requires problem and context knowledge of the procedure to collect compatible data. If the dataset contains sufficient and adequate data and information, problems such as overfitting and underfitting could be mitigated. Furthermore, the usage of multiple available datasets is of paramount importance to obtain a more reliable benchmarking of existing and new methods. In this section, the publicly available datasets for different categories of image forensics tasks will be introduced. Different datasets are grouped according to the different image forensics categories for which they are used.

### 2.1. Original Data

Datasets of pristine data used in the image forensics field (e.g., in the manipulation detection area) often contain original uncompressed image data. In this way, researchers can recreate different manipulation operations and conduct experiments on an adequate and customized dataset. Some of these databases were originally designed for the purpose of benchmarking camera identification techniques.

One of the first works in this field is the UCID dataset [15] with 1338 uncompressed images (version 2) in TIFF format stemming from a single camera. The BOSSBase 1.01 dataset [16] contains 10,000 grayscale uncompressed images, originally designed for research in the steganalysis field. In the Dresden image dataset [17], 73 digital cameras with 25 different models were used to create 14,000 Joint Photographic Experts Group (JPEG) images. The RAISE dataset [18] contains 8156 uncompressed high-resolution images of different categories such as landscape or indoor scenes. It comprises 4 subsets called RAISE-1K, RAISE-2K, RAISE-3K and RAISE-4K.

Some recent datasets introduced cell phone cameras to their catalogue. A small number of devices was considered in the MICHE-I dataset [19] comprising 3732 iris images from 3 different smartphones using both front and back cameras. The IEEE and Kaggle [20] organized a camera identification challenge in 2018 with a dataset captured from 10 different camera models (9 of 10 being smartphone cameras) with 275 images from each device.

The Vision dataset [21] also purposed for camera model identification and contained 34,427 images and 1914 videos from 35 portable devices of 11 major brands, both in the native format and in their social platform version including Facebook, YouTube and WhatsApp. Some datasets like [22,23] are designed for a specific domain. For instance [23] is an ongoing collection of satellite images of all land on Earth produced by the LandSat 8 satellite. Other proposals like [24,25,26,27,28], initially designed for object and scene detection, segmentation and recognition, were used in the image forensics field to create synthetic data. For example, the Microsoft COCO dataset [25], originally constructed for object and scene analysis and comprising more than 300,000 images in JPEG format, has been used to create different image forgeries. Another example is the SUN2012 dataset [28], composed of 130,519 images of different outdoor and indoor scenes, has been employed to create synthetic data for image forensics purposes.

Regarding the creation of Deepfakes (i.e., fake images generated by deep neural networks), some well-known datasets of human faces have been used for network training, for instance the CelebA dataset [29] which contains around 200,000 faces with different annotations originally designed for facial image analysis. Stemming from CelebA dataset, CelebAHQ [30] is a set of high-resolution face images and is one of the first datasets used for training and evaluation of Generative Adversarial Network (GAN) models for face generation and editing.

### 2.2. Falsified Data

To our knowledge, the first public datasets for detection of *splicing* (i.e., a common image falsification in which one copies a part of an image and pastes it to another image) were the Columbia gray DVMM dataset [31] and the Columbia color splicing dataset [32]. The two datasets comprise respectively 1845 grayscale images for the first one and 180 color spliced images for the second one, both with rather non-realistic random-like splicing falsifications. Two other well-known splicing datasets are the CASIA V1 and V2 [33] with more realistic forgeries and post-processing operations on the V2 to cover the traces of splicing. In 2013, the IEEE Information Forensics and Security Technical Committee (IFS-TC) organized an image forensics challenge and released a dataset of pristine and forged images [34] with a set of different falsification techniques such as splicing and *copy-move* (i.e., another common falsification in which one copies a part of an image and pastes it in the same image). Each fake image had an associated ground-truth binary map showing the regions that were falsified. As a small sub-dataset from the IFS-TC proposal, the DS0-1 dataset (also known as Carvalho dataset) [35] contains forgeries created in a careful and realistic manner.

**Figure 3:**
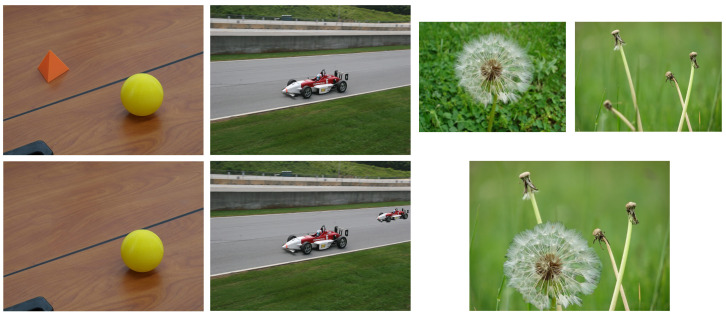
Sample images from the NIST Nimble 2016 Dataset [36]. Top row shows the original images, and bottom row shows from left to right falsifications of inpainting-based removal, copy-move and splicing.

The National Institute of Standards and Technology (NIST) developed the Nimble [36] and MFC [37] datasets. The first one, often called NIST Nimble 2016, included three types of falsifications including splicing, copy-move and *inpainting* (i.e., a third type of common falsification in which a part of an image is replaced and filled with realistic synthetic content), with different levels of compression and post-processing. Figure 3 shows some example images from this dataset. The NIST MFC17 dataset [37] included more challenging image forgeries but did not contain the different compressed versions.

The Realistic Tampered Dataset [38], also known as Korus dataset, comprises 220 splicing and copy-move forgeries of a realistic level. The authors included PRNU signatures and ground-truth maps. Other datasets have been created with a specific purpose in mind. Regarding the double compression scenario, the VIPP dataset [39] was created to evaluate the detection of double JPEG compression artifacts which may be present for instance in the splicing falsification.

The use of datasets specific for copy-move falsification, such as [40,41], is not very common for the deep-learning-based detection methods. The main reason is that existing datasets are relatively small. Therefore, majority of research on deep-learning-based copy-move detection has created customized synthetic datasets which are derived from dataset of original images and which contain much more samples.

Table 1 shows a list of popular datasets for image forensics research including datasets of original data and falsified data. In the case of falsified data, we provide the number ratio of pristine and tampered-with images. Regarding the “Operations” columns we mention the main operations (mostly falsifications) contained in the dataset and the “Others” case mainly includes double JPEG compression.

### 2.3. Artificially Generated Data

In the case of artificially generated data, it is important to use datasets that contain realistic examples. Existing datasets considered different scenes of authentic images taken by a camera and artificially generated fake images either with conventional Computer-Generated Image (CGI) creation algorithms or recent GAN architectures.

One of the first popular dataset of CGIs is the Columbia dataset [43] with 1600 photorealistic computer graphics images. Afchar et al. [44] created a dataset with 5100 fake images generated from videos downloaded from the Internet. Rahmouni et al. created a dataset of CGIs coming from high-resolution video game screenshots. There are several online repositories for CGI [45,46,47,48] that have been used as datasets for different detection approaches.

A small dataset of 49 Deepfake and 49 original videos was created by Yang et al. [49] using the FakeApp application. A bigger one is the FaceForensics dataset [50] comprising about 1000 videos and their corresponding forged versions focused on expression swap created with the Face2Face model. The same authors extended the dataset [51] with 4000 forged videos. Li et al. [52] created a dataset of 590 original videos and 5639 Deepfake videos. In comparison to other face datasets [29,30], the diversity among genders, ages and ethnic groups is bigger. The IDIAP institute created DeepfakeTIMIT [53] also known as DF-TIMIT containing 620 videos where faces were swapped. This dataset was generated using the faceswap-GAN [54] with 32 subjects and 2 subsets of different resolutions: low quality with 64×64 and high quality with 128×128.

Recently, Google in collaboration with Jigsaw and Facebook have created a Deepfake dataset to contribute to the relevant research. In 2019, Facebook created the DFDC dataset [55] for the Deepfake detection challenge with 4113 Deepfake and 1131 original videos from 66 subjects of diverse origins who gave their consent for the relevant data. Finally, the DFD dataset [56] contains 3068 Deepfake videos and 363 original ones from 28 individuals who consented to the data.

Table 2 summarizes the artificially generated datasets presented above. The “Content ratio” column shows the number of pristine/fake images.

From the next section, we present different kinds of deep-learning-based image forensics methods, starting by the detection of routine image manipulations.

## 3. Manipulation Detection

We consider image manipulation as routine operations modifying or improving digital images with basic and benign image processing such as median filtering, JPEG compression or contrast enhancement. These operations may be used to enhance the visual quality of tampered-with images or to hide the traces of falsification operations that may leave an apparent fingerprint if used alone. In this subsection we introduce the most relevant strategies to detect some of the most common manipulation operations using deep learning as the core technique. We present both *targeted* (i.e., aiming at a specific manipulation operation) and *general-purpose* (i.e., aiming at various operations) detectors.

### 3.1. Median Filtering Detection

The early deep-learning proposals in the literature of image forensics were focused on designing a specific strategy to cope with each forensic challenge individually. The goal of one of the first methods proposed in 2015 by Chen et al. [58] was to detect median filtering manipulation.

In their paper, Chen et al. [58] used a tailored Convolutional Neural Network (CNN) to detect median filtering with JPEG post-processing. The JPEG compression after median filtering made the forensic problem more challenging because the compression can partially remove the forensic traces of medial filtering. The tailored CNN took the Median Filtering Residual (MFR) rather than the raw pixel values as input for the first layer in the CNN. The MFR is the difference between a given image and its median filtered version. The authors found that using this special input, the network achieved a better forensic classification performance, with a better detection accuracy when compared with handcrafted-feature-based strategies on small patches of 64×64 and 32×32.

More recently, Tang et al. [59] proposed to upscale the input with nearest neighbor interpolation in an attempt to enlarge the difference between manipulated and original patches. After this upscaling, the first two layers in the network are mlpconv layers introduced in [60]. An mlpconv consists of a special layer for deep-learning architectures that defines a group of convolutional layers with activation functions that can enhance the non-linear ability of the network. Specifically, it proposes to replace a traditional convolutional layer followed by a Rectified Linear Unit (ReLU) activation function with a convolutional layer, ReLU activation function, convolutional layer with filters of shape 1×1 and a final ReLU activation function. In the case of median filtering detection, Tang et al. [59] made use of mlpconv to enhance the network’s non-linearity to deal with the detection of median filtering non-linearity.

Both the above proposals [58,59] rely heavily on having a special input for the network being either the MFR or an upscaled version, regardless of their differences in the network architecture.

### 3.2. Double JPEG Compression Detection

JPEG images are widely used in daily life as one of the most common image formats. Hence, most of the forensic tasks are related to JPEG images. Typically, inside a normal forgery creation process, an image is decompressed from JPEG to the spatial domain for falsification, and later recompressed again in JPEG format for storage and further use. For this reason, the image forensics community has dedicated important research efforts to the detection of double JPEG compression through the years. Detection and localization of double JPEG compression provides valuable information towards image authenticity assessment.

In double JPEG compression, double quantization of Discrete Cosine Transform (DCT) coefficients leaves special artifacts in the DCT domain, in particular, on histograms of block-DCT coefficients [61]. In [62,63] authors proposed to use as input the concatenation of DCT histograms for their CNNs. These approaches outperformed non-deep-learning methods, especially on small-sized images up to 64×64 pixels. Afterwards, Barni et al. [64] found that CNN architectures could detect double JPEG compression with high accuracy when the input of the network was noise residuals or histogram features; this was tested on double compression with both different and same quantization matrix.

**Figure 4:**
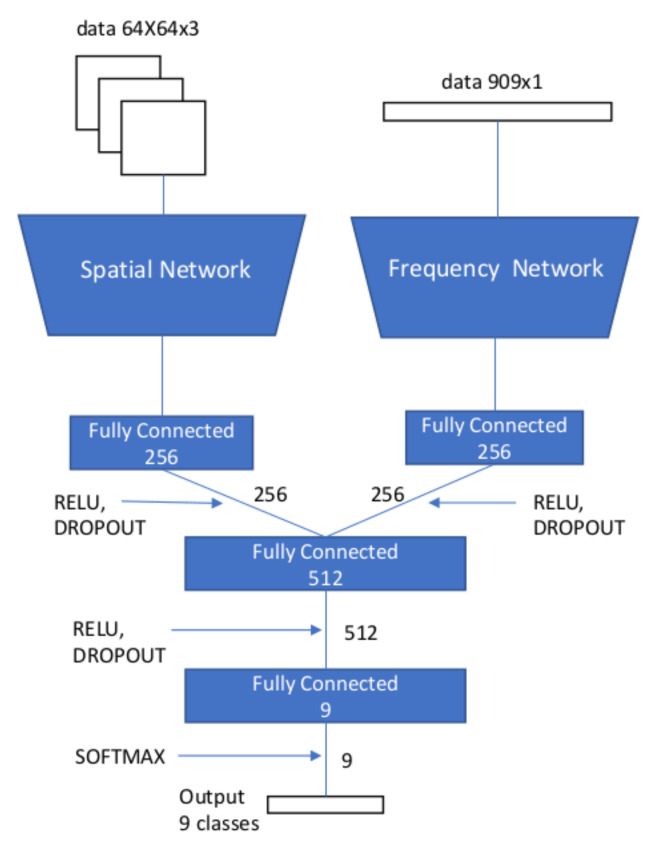
Architecture of the multi-domain convolutional neural network proposed in [65] for double JPEG compression detection.

In [65], Amerini et al. designed a multi-domain convolutional network to detect and localize double JPEG compression. They proposed to use both DCT coefficients and spatial features for the localization. The architecture achieved a better detection accuracy when compared to using only pixel values or DCT coefficients. In their implementation, two branches were used as inputs for the network, one receiving the image patches and the other the DCT coefficients. After several convolutional blocks (convolutional layer, activation function and pooling layer), both outputs are concatenated and fed to a final fully connected layer followed by the classification layer for detecting different JPEG quality factors. Figure 4 shows the proposed multi-domain neural network. The architecture of the sub-network with the frequency-domain input has some similarities to the one in [62], while the range of the bins in the DCT histogram is augmented.

The method proposed in [66] extracted block-wise histogram-related statistical features under mixed quality factor conditions to achieve better accuracy and localization capability. The proposed CNN takes a multi-branch approach using histogram features and quantization tables as inputs. The quantization branch is directly concatenated to the last max-pooling layer output and two fully connected layers. The authors reported that the ability of the network to distinguish between single and double JPEG compressed blocks was dramatically improved by including quantization table branch.

The above presentation suggests that using special features as input for the first layer of CNN can achieve good detection performance and that in the case of using multiple inputs the multi-branch approach can combine them properly.

### 3.3. Contrast Enhancement Detection

Like median filtering, contrast enhancement is one of the routine image manipulations commonly applied to conceal the traces of tampering. In the case of a falsified image, it is common to have contrast differences between the background and the forged region, which may be caused by different lightning conditions. In this scenario, contrast enhancement is broadly used to remove or alleviate visual clues that would give away the forgery. Consequently, detecting the application of this operation has drawn researchers’ attention in the image forensics field [67].

In [68] authors proposed a 9-layer CNN that is directly fed with 64×64 image pixel values with no special features, making the discriminative features self-learned by the network. The authors showed good robustness against JPEG compression post-processing over a wide range of quality factors by training the network with different contrast adjustments. The proposed architecture also generalized well to unseen tonal adjustments.

Sun et al. [69] proposed to use the Gray Level Co-occurrence Matrix (GLCM) which is computed by accumulating the occurrence of the pixel intensity pairs between each pixel and its neighboring pixels. The GLCM was used as input for a shallow CNN of three convolutional groups for detecting contrast enhancement. The authors reported good results when an image is JPEG compressed after the contrast enhancement on 256×256 image patches. The proposed method outperformed the conventional ones in terms of the manipulation detection accuracy.

Using the GLCM as input of the network in a similar way, Shan et al. [70] also proposed a JPEG-robust forensic technique based on CNN to detect both local and global contrast enhancement. The adopted network architecture is one convolutional block (4 layers in one block) deeper than the one proposed in [69]. Experimental results showed that Shan et al.’s method could efficiently detect both local and global contrast enhancement in compressed images regardless of the order of contrast enhancement and JPEG compression.

### 3.4. General-Purpose Manipulations Detection

The manipulation detection methods presented until now focus on the detection of a specific and targeted manipulation. This limits the application range of such methods because for creating a doctored image, several different processing operations can be applied to obtain a visually convincing result. For instance, in the case of splicing falsification, the forged part of the image can go through one or several basic operations such as rescaling, contrast enhancement and median filtering. Therefore, it is of great importance to develop general-purpose strategies that can detect different kinds of image manipulation operations.

As mentioned in previous subsections, the usage of special features in the CNN input in general leads to a better performance for image forensics problems. Following this approach, Bayar and Stamm [71] proposed a new constrained filter for the first layer of a CNN to suppress the image content for detecting various image processing operations. Their constrained network is forced to learn a set of high-pass filters by imposing a constraint on the weights of all the *K* first-layer filters in each forward pass of the learning process. This filter constraint enforcement is shown in the following Equation (1) as proposed in Bayar and Stamm’s original paper [71]:

(1) $$\left\{\begin{array}{c} w_{k}^{\left(1\right)}\left(0,0\right)=-1,\\ \sum_{m,n\neq 0}w_{k}^{\left(1\right)}\left(m,n\right)=1, \end{array}\right.$$

where $w_{k}^{\left(1\right)}\left(m,n\right)$ denotes the weight at position (m,n) of the *k*th filter in the first layer (the indices *m* and *n* can be negative or positive), and $w_{k}^{\left(1\right)}\left(0,0\right)$ denotes the weight at the center of the corresponding filter kernel. In this manner the sum of all filter elements in each filter is 0, and the constrained first-layer filter operates like a high-pass one by effectively removing image content. This prediction error layer extracts and highlights the local dependency of pixels with its neighbors, which is an important piece of information from the forensics point of view. Experimental results in [71] also showed that the usage of tanh as activation function outperforms the more common functions such as ReLU. The reason may be that tanh tends to preserve more information related to the sign of the values at the function input, without setting all negative values to be 0 as in ReLU. The sign information may be important for image forensics tasks.

Recently, Castillo Camacho and Wang [72] proposed a different initialization method for the first layer of a CNN to cope with a challenging setting of general-purpose image manipulation detection. It is challenging because the considered manipulations are of small amplitude. Taking advantage of the milestone work of the famous Xavier initialization [73], they proposed a way to create random high-pass filters that could operate without constrains. The method had a high detection rate for manipulations such as median filtering, Additive White Gaussian Noise (AWGN) and resampling. Recently, the same authors [74] proposed a data-dependent scaling approach for first-layer filters initialized by different algorithms. The proposed approach considered natural image statistics and could ensure the stability of the amplitude (i.e., the variance) of data flow in a CNN, which was beneficial for general-purpose image manipulation detection.

### 3.5. Summary and Comparisons of Manipulation Detection Methods

Besides qualitative comparisons between different forensic methods (in particular special network design and special input features), we have also made efforts to carry out quantitative comparisons of forensic performance for each category of methods. In order to ensure as much as possible a fair comparison, performances are extracted from the original papers and reported for the most commonly used databases whenever possible. Concerning the cases where the results for several patch sizes are available, we share the results for the most common size among the compared methods.

Regarding the metric used for evaluating the forensic performance, we have endeavored to select the most common one among each category of methods. We mention the metric used for each method when we are forced to use different metrics for different methods even on a same database. Indeed, given the heterogeneous experimental settings adopted in the original papers, it is in general difficult to carry out performance comparison that can cover all methods with a same setting of tested database and used metric.

The most commonly used metrics in this review are accuracy, precision (mainly the average precision as defined later in this paper), and Area Under the Curve (AUC) score. Accuracy is the percentage of correctly classified samples among all samples, as calculated with the following equation:

(2) $$\mathrm{Acc}=\frac{\mathrm{TP}+\mathrm{TN}}{\mathrm{TP}+\mathrm{TN}+\mathrm{FN}+\mathrm{FP}},$$

where TP, TN, FP, and FN stand for respectively true positive, true negative, false positive and false negative numbers of classified samples.

The precision represents the fraction of correctly classified positive samples among all samples classified as positive, which is computed as

(3) $$\mathrm{Prec}=\frac{\mathrm{TP}}{\mathrm{TP}+\mathrm{FP}}.$$

Theoretically, the AUC score is equal to the probability that a model ranks a randomly selected positive sample higher than a randomly selected negative one. It is defined by the following formula

(4) $$\mathrm{AUC}=\int_{0}^{1}\;\mathrm{TPR}\left({\mathrm{FPR}}^{-1}\left(x\right)\right)dx,$$

where the true positive rate is defined as TPR=TP/(TP+FN) and the false positive rate is defined as FPR=FP/(TN+FP). In practice, in order to obtain the AUC score which lies between 0 and 1, we first draw the Receiver Operating Characteristic (ROC) curve of TPR against FPR for a classifier by varying the decision threshold, and then we compute the area under this curve.

The choice of metric depends on many factors, including the forensic problem at hand, experimental setting, a kind of tradition among researchers working on a same problem, preference of authors of a forensic method, and technical or even legal requirements when a forensic detector is deployed in real-world applications. For instance, in an experimental setting with imbalanced data from different classes, the accuracy metric in general results in biased value and thus is not preferred; in certain application scenarios, we need to consider a decision threshold corresponding to a certain level of false positive rate; etc. Nevertheless, in academic papers, authors often consider a simplified and controlled laboratory experimental setting and accordingly attempt to achieve good performance in terms of an appropriate metric of their choice. As mentioned earlier, in this review for a fair comparison we extract and report results from the original papers of forensic methods. When comparing a category of methods, we try to use the most commonly adopted metric among different papers and intuitively explain why this metric is used. However, in many cases we are forced to report results in terms of different metrics as adopted in the original papers of compared methods. Indeed, it would be important that the research community could build high-quality benchmarking datasets with a unified metric for each dataset (e.g., with instructions on how to choose decision threshold).

Table 3 summarizes existing deep-learning-based image manipulation detection methods, by considering different technical aspects in particular the input feature of the network and the specificity of CNN design. Listed methods created an ad-hoc dataset of manipulated images/patches from pristine images. We show in the table the original datasets of pristine images used to create manipulated samples for each method. Meanwhile, every method may also have its own parameters of manipulation operations, e.g., different JPEG compression quality factors. Consequently, performance of each method is shown on an ad-hoc dataset, except for the comparison of general-purpose manipulation detection methods (i.e., the group of methods named GIPO in Table 3). For the comparison of GIPO methods, we report the performance results extracted from [74] where a fair comparison was conducted by using same datasets of pristine and manipulated images/patches and same manipulation operations with same parameters. We can observe from Table 3 that the most commonly used metric is the accuracy. This is mainly because of the fact that for image manipulation detection researchers almost always consider a controlled laboratory experimental setting with balanced data from each class. Therefore, in this case the accuracy is a simple and adequate metric that has been widely used by researchers working on manipulation detection. In the following, we present and analyze each group of methods.

Median filtering comparison results were taken for a patch size of 64×64 with a median filter kernel of size 3. Slightly different datasets of pristine images were used in the two compared methods [58,59]. From the results in Table 3 we can see that Tang et al.’s method [59] obtained better results, which implies that upscaling can be an effective pre-processing for the detection of median filtering by using CNN. This pre-processing would make the traces of median filtering more prominent and easier to be detected by a neural network.

The double JPEG compression detection methods used different datasets and settings for their experiments. In some methods the quality factor for each compression was taken randomly from a uniform distribution while other methods used pre-defined fixed factors. Additionally, in some cases aligned double JPEG compression was considered while this point was omitted or not clearly presented in some other methods. Nevertheless, we present the best case shared by each method when tested on patches of size 64×64. The best case varies for different methods, for example for the method in [65] the best case was obtained with a first compression with quality factor of 55 followed by a second compression with quality factor of 85, while for the method of [62] it was achieved with quality factor of 70 and 80 for respectively the first and the second compression. We are aware that the results are not directly comparable, and our purpose here is to give a rough idea on the performance of forensic methods designed to detect double JPEG compression. From the results in Table 3, we can observe that the performances of all methods in the best case are quite satisfactory, all higher than 90%. Interestingly, all methods considered DCT features as network input. These features appeared to be effective in detecting double JPEG compression manipulation, and this may intuitively explain the good performance of all compared methods.

The papers on contrast enhancement detection also used different databases and experimental settings for the validation of their methods. In Table 3, results are provided for gamma correction with factor 0.6 and a random value taken from {0.6,0.8,1.2,1.4}, on patches of size 64×64 and 256×256, respectively for the methods proposed in [68,70]. The experimental setting of [69] was more complicated: the result shown in the table was obtained on a dataset of 256×256 patches manipulated with a combination of three different contrast enhancement techniques being histogram stretching, gamma correction (with a factor randomly taken from {0.5,0.8,1.2,1.5}), and S-curve mapping. Though it is not easy and not our purpose to rank the performance of the three methods mainly due to different experimental settings, all of them achieved very good results close to a perfect detection of 100%. This may imply that CNN is able to extract discriminative information from both pixel values [67] and GLCM features [68,69] for detecting contrast enhancement.

The comparison of general-purpose manipulation detection methods is made for 64×64 patches with results taken from [74]. A challenging experimental setting with five different manipulations (median filtering, Gaussian blurring, additive white Gaussian noise, resizing, and JPEG compression) was tested for the three methods under comparisons [71,72,74]. As mentioned above, same datasets and same manipulations with same parameters were used for each method to ensure a fair comparison. From the results in Table 3, we can see that the method in [74] outperforms the two other methods. This is because [74] attempts to keep a stable data flow for the first convolutional layer which normally has a special design. This means that a combination of an appropriate design of first-layer filters (e.g., high-pass filters) and a proper scaling of these filters can lead to a better performance.

## 4. Falsification Detection

We consider image falsification as the creation of fake content in some part of the image to deceive viewers about the facts happened in the past. In contrast to routine image manipulation, image falsification is conducted intentionally to change the image’s semantic meaning, often by inserting or removing certain content.

The most common image falsification techniques can be roughly divided into three broad categories: copy-move forgery where one part of the image (the source region) is copied and pasted into the same image as the fake part (the target region); splicing forgery where the tampered-with region in a host image was originally from a different image; and inpainting forgery which is sometimes considered to be a subgroup of copy-move with the difference that the fake region in inpainting falsification is often constructed by using and combining small motifs at different locations of the same image. It is worth mentioning that the inpainting technique is traditionally used to reconstruct a lost or corrupted part of the image and that inpainting falsification is often applied for carrying out object removal in an image. Research on splicing detection is in general more active than copy-move and inpainting. This is probably because it is more convenient to create diverse splicing forgeries from a large pool of publicly available pristine images. Figure 3 shows, from left to right, examples of inpainting, copy-move and splicing forgeries. In the following, we will organize the presentation of deep-learning-based falsification detection methods into two groups: (1) *multipurpose* detectors which can detect different kinds of image forgeries among the above three categories and (2) *targeted* detectors which are focused on the detection of one specific falsification.

### 4.1. Multipurpose Detectors

Multipurpose detectors are usually based on the general assumption that any image falsification introduces statistical deviation with respect to the authentic part, i.e., within the fake region, around the fake region boundary, or both.

Zhang et al. [75] proposed to use an autoencoder [76] which is a type of neural network taking an image as input and reconstructing it using fewer number of bits. Wavelet features were used as input for the network to detect and localize in a patch-wise manner the tampered-with regions. Besides wavelet features, local noise features originally proposed for steganalysis, such as Spatial Rich Model (SRM) [77], have been largely used to solve image forensics problems with encouraging results. In SRM, a group of handcrafted filters was designed to extract local noise from neighboring pixels, and this often allows us to obtain disparities between forged and original areas. SRM filters have been used for creating a special input for CNNs. This is one important difference from CNNs used in computer vision tasks: it is considered beneficial for CNNs of image forensics tasks to use SRM filters as initialization for the first layer, instead of the random weights conventionally used in CNNs from the computer vision community. In [78], Rao and Ni proposed to use the 30 SRM filters as initialization for the first layer in a CNN to detect splicing and copy-move forgeries. The results from the pre-trained CNN were used in a Support Vector Machine (SVM) classifier for solving a binary problem (authentic/forged). In a similar approach based on steganalysis features, Cozzolino et al. [79] proposed to use a shallow or short CNN to detect image forgeries on small patches.

**Figure 5:**
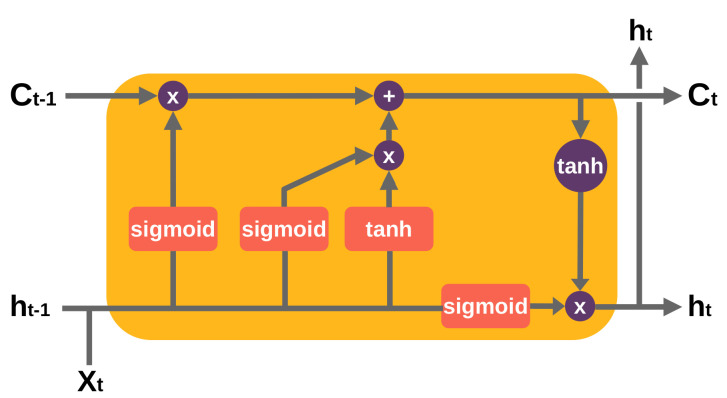
An LSTM cell. $X_{t}$ is the input, $h_{t-1}$ and $h_{t}$ are the output of the previous and current block, $C_{t-1}$ and $C_{t}$ are the cell state of the previous and current block. An LSTM block can help to correlate neighboring blocks and search for inconsistencies when a forgery is present. This is achieved via gates of activation functions to determine if certain data is relevant for forwarding it or forgetting it.

In [80,81], authors made use of a Long Short-Term Memory (LSTM) architecture for localizing at pixel level the tampered-with regions. An LSTM as proposed in [82] is a special type of Recurrent Neural Network (RNN) designed for sequences or time series data. An LSTM layer consists of a set of recurrently connected blocks, known as memory blocks. Each block contains one or more recurrently connected memory cells and three multiplicative units—the input (sigmoid and tanh functions), output (sigmoid and tanh functions) and forget (a sigmoid function) gates—that regulate the flow of information into and out of the cell. Figure 5 shows an unrolled example of an LSTM block. The core strength of using LSTM in the image forensics field is to acquire from previous blocks the boundary information, which is decisive to obtain particular features to classify between original and tampered-with regions. In [80] experiments showed that both CNNs with Radon transform as input and LSTM-based strategies were effective in exploiting resampling features to detect and localize tampered-with regions. Bappy et al. [81] proposed an LSTM and an encoder–decoder network to semantically segment falsified regions in a tampered-with image.

Some researchers suggested that a CNN trained for detecting camera traces could be used to detect and localize image splicing. If an analyzed image contains patches of different sources, then the blocks can be clustered in different groups separating the suspicious area. Works in [83,84] made use of camera-specific features obtained by a CNN that focuses on them. Both methods analyzed patches and looked for traces of different cameras in the same image. Bondi et al. [83] used a clustering algorithm to create different groups of the authentic and suspicious areas. In [84] a noise residual called *Noiseprint* was extracted and used to check inconsistencies within a single image.

Yarlagadda et al. [85] used a GAN that included an adversarial feedback loop to learn how to generate some information in a realistic manner, with the objective to detect satellite image forgeries. There are two major components within GANs: the *generator* that takes a noise vector as input and outputs an image improved at each step with the knowledge of what a valid input should be, and the *discriminator* that tries to classify between real and fake (i.e., created by generator) content. Their proposed architecture was followed by an SVM to detect whether feature vectors come from pristine images or forgeries.

**Figure 6:**
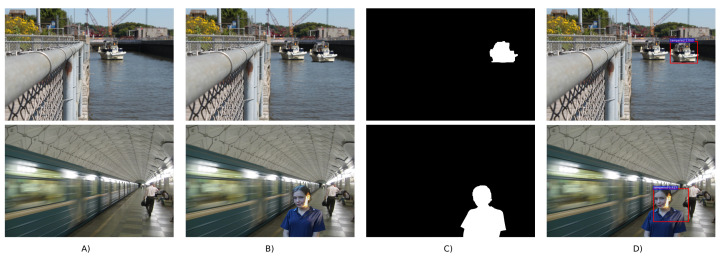
Bounding-box localization results generated by using the implementation of [86] on NIST 16 dataset [36]. Top and bottom rows show copy-move and splicing examples, respectively. (**A**) is the original image, (**B**) is the falsified image, (**C**) is the ground-truth mask, and (**D**) is the localization result.

Recently, refs. [86,87] proposed the multi-branch CNNs to tackle the challenge of image forgery detection. Specifically, Zhou et al. [86] proposed a multi-branch Region-Convolutional Neural Network (R-CNN) which is a type of CNN typically used for object detection to coarsely locate the tampered-with regions in bounding boxes. The authors used pixel values in one branch with ResNet-101 architecture [88] and noise features obtained by SRM filters in the second branch. Wu et al. [87] suggested a multi-branch CNN joined with an LSTM trained with a set of 385 different image manipulations. Their architecture named Mantra-Net generates a pixel-level detection mask reflecting the probability of a falsification. In the three input branches of Mantra-Net the first layers are initialized with SRM filters, high-pass constrained filters of Bayar and Stamm [71], and normal random weights. Figure 6 shows example results of bounding-box localization of falsifications produced by Zhou et al.’s detector [86].

Very recently, Mara et al. [89] worked on a full-image CNN based on Xception architecture [90] to detect and localize image falsifications. The proposed end-to-end network used the *Noiseprint* [84] as features extracted from the image input. Meanwhile, in [91] a GAN was proposed to generate falsified images avoiding the burdensome task of creating and labeling image forgery examples in a conventional way. With this big number of synthetic examples, the proposed algorithm was able to segment and refine the focus on boundary artifacts around falsified regions during the training process.

Table 4 provides a summary of the various multipurpose falsification detection techniques. The summary includes the method reference, input for the network, initialization used in the first layer, input size, localization level, considered databases, and network type. We also show in the last two columns of the table the performance comparisons on the two most common datasets used among all methods, i.e., CASIA [33] and NIST 16 [36]. Besides the accuracy metric and the AUC metric, respectively introduced in Equations (2) and (4) in Section 3.5, in the table we also use a new metric of F-1 score which is defined by the following equation:

(5) $$F1=\frac{2\mathrm{TP}}{2\mathrm{TP}+\mathrm{FP}+\mathrm{FN}}.$$

In Table 4, the reported results correspond to patch size of 64×64 and 256×256 for [80,87], respectively. For the other methods, the performance corresponds to the only patch size given in the column of “Input size”. It is worthwhile mentioning that the performance is reported at image level for [78] and at patch level for [75], while for all other methods the metric is pixel-level localization performance which is naturally a more challenging metric than image-level and patch-level counterparts. We can observe from Table 4 that besides the accuracy, the AUC and the F1-score have also been used as performance evaluation metrics. This is probably because for falsification detection researchers usually have imbalanced classes of authentic and falsified samples, with the falsified samples being fewer than the authentic ones. In this case of imbalanced classes, the AUC and the F1-score are more appropriate metrics than the accuracy. On the CASIA dataset the methods of [86,87] achieve satisfying performance of pixel-level localization results. We notice that both methods have either a special input of noise features [86] or a special design of first-layer filters [87]. It appears that both options can be effective in detecting and locating falsifications which may leave traces in the high-frequency component of images. On the NIST 16 dataset, methods of [80,81] share comparable and very good results. These two methods consider resampling traces as one of the discriminative features for the falsification localization task. This technical choice seems quite adequate for exposing forgery traces on falsified images in the NIST 16 dataset.

### 4.2. Targeted Detectors

Targeted detectors, which are designed to detect only one type of image falsification, have been developed in parallel with multipurpose ones.

#### 4.2.1. Splicing Detection

Some early works dealing with splicing detection and localization were based on autoencoders. In [93], authors used SRM features as input for their autoencoder model. The method in [94] used the steganalysis features from SRM to analyze frames in a video with autoencoder and LSTM to detect splicing forgeries.

Wu et al. [95] proposed a framework of Constrained Image Splicing Detection and Localization (CISDL) based on the well-known VGG-16 architecture [96]. Using two input branches they calculated the probability that one image had been partially spliced to another one and localized the spliced region. Meanwhile, in [97,98], a CNN without fully connected layers known as Fully Convolutional Network (FCN) [99] was used to predict a tampering map for a given image. In [97], the proposed architecture has two exit localization branches. The first one was used for localizing the inner part of the spliced area and the second one for detecting the boundary between pristine and spliced regions. Concurrently, Liu et al. [98] made use of three FCNs to deal with different scales; moreover, conditional random field was used to combine the results of different scales.

Some approaches [100,101] attempted to detect anomalies or inconsistencies within tampered-with images. In [100], a Siamese CNN with a self-consistency approach to determine if contents had been produced by a single device was proposed. The proposed model could predict the probability that two patches had similar EXchangeable Image File (EXIF) attributes and output a “self-consistency” heatmap, highlighting image regions that had undergone possible forgery. In [101] authors used transfer learning from a pre-trained residual network (ResNet-50) with illumination maps taken from input images to find hints of forgeries.

Recent strategies [102,103] made use of U-Net [104] architectures. In a U-Net, the features are captured by a size-reducing way of consecutive layers, then upsampled and concatenated with the first path in a *U*-shaped symmetric path, attempting to reduce loss and improve localization capability. In [102], authors took advantage of U-Net architecture for the training of a GAN with image retouching generator, which helped a splicing localization model to learn a wide range of image falsifications. Meanwhile Bi et al. [103] proposed a method mainly based on U-Net as a segmentation network for splicing forgery detection.

Given the popularity of GANs in the computer vision field, some researchers have also started to use them for image forensics purposes. This is the case of [105] where the authors made use of a conditional GAN for the training of a detector to locate forgeries in satellite images. Liu et al. [106] proposed a deep matching CNN together with a GAN to generate probability maps in a CISDL scenario.

Special initialization of first layer was also considered for splicing detector. For example, Rao et al. [107] designed and implemented a CNN with the first layer of the network initialized with 30 SRM filters to locate splicing forgeries.

Table 5 summarizes the targeted detectors of splicing falsification. The considered properties of the detection methods are similar to those in Table 4. A performance comparison on the two most common datasets used among all methods (i.e., Carvalho [35] and CASIA [33]) is provided, in terms of pixel-level falsification localization performance. Besides the accuracy and F-1 score metrics which were introduced previously, in the table we use a new metric of mean average precision (mAP). In order to define this new metric, we first introduce the definition of the average precision (AP) metric as shown in the following equation:

(6) $$\mathrm{AP}=\int_{0}^{1}\;\mathrm{Prec}\left(r\right)dr,$$

where Prec is the precision metric as given in Equation (3) and *r* is the recall metric defined as $r=\mathrm{Recall}=\frac{\mathrm{TP}}{\mathrm{TP}+\mathrm{FN}}$. In practice, the precision can be regarded as a function of the recall when varying the decision threshold, and vice versa. The AP metric calculates the average precision value $\mathrm{Prec}\left(r\right)$ for recall value *r* varying from 0 to 1. Consequently, the mAP metric is defined as the mean average precision over all classes, as given by:

(7) $$\mathrm{mAP}=\frac{1}{C}\sum_{i=1}^{C}\mathrm{AP}\left(i\right),$$

where *C* is the number of classes and *i* represents a particular class. In Table 5, forensic performance is reported in terms of F1-score and mean average precision, mainly for two reasons: (1) these two metrics are well suited for the classification problem of imbalanced classes of authentic and falsified samples; (2) the mean average precision has been introduced probably by researchers who have worked for long time in the computer vision field where the mAP is a widely used metric.

We notice from Table 5 that performance on Carvalho dataset is rather limited for existing methods. As mentioned in Section 2.2, falsified images in Carvalho dataset were carefully created. This limited performance implies that forensic analysis of high-quality falsified images is still a challenging task, and future efforts shall be devoted to this research problem. By contrast, falsified images in CASIA dataset are less difficult to handle. Recent methods achieved good results on this dataset, either by leveraging adversarial learning [106] or by using special forensic features as network input [107].

#### 4.2.2. Copy-Move Detection

Copy-move detection is one of the forensic techniques that have been studied with more balance between conventional and deep-learning approaches. As mentioned before, in a copy-move forgery, a part of the original image (source area) is copied and pasted at a different place (target area) of the same image. Before pasting, the target area can be transformed (rotation, scaling, shearing, etc.) to make the forgery visually realistic. Routine image manipulation (smoothing, contrast adjustment, etc.) can be applied locally or globally to enhance the visual quality. Copy-move is mainly used for falsifications where certain content needs to be disguised or cloned.

Probably the first proposal using a deep-learning approach to solve the copy-move detection problem was the method from Ouyang et al. [108], which was based on the famous pre-trained AlexNet [109] originally designed for image recognition. The authors generated forged images by choosing a random square from the upper left corner and copying it to the center. Although this method obtained decent results in this artificial scenario, the performance was diminished for realistic forgeries.

**Figure 7:**
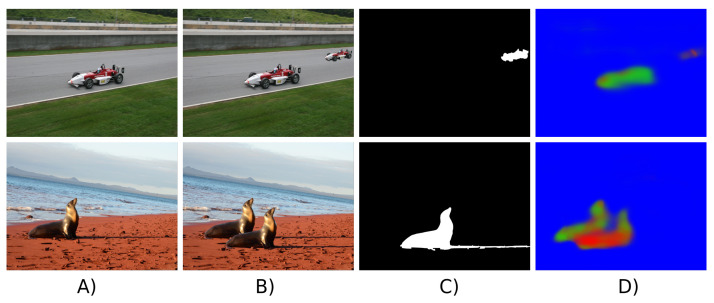
Source-target disambiguation results generated by using the implementation of [111] on images from the NIST 16 dataset [36]. (**A**) is the original image, (**B**) is the falsified image, (**C**) is the ground-truth mask, and (**D**) is the localization result in which green and red color represents respectively the source (original) and target (falsified) region in a copy-move forgery.

Wu et al. [110] proposed a CNN-based method which first divided the input image into blocks, then extracted special features, correlated features between blocks, localized matches between blocks and finally predicted a copy-move forgery mask. Furthermore, routine image manipulation operations to hide the forgery traces such as JPEG compression, blurring and AWGN were applied to training data as a means of data augmentation. The objective was to easily detect these manipulations as possible telltales of copy-move falsification. Very shortly after this piece of work, the same authors [111] proposed to use a different architecture with two exit branches to deal with the problem of source-target disambiguation where it is necessary to discern between source (original) and target (falsified) regions in a copy-move forgery. Another deep-learning method for source-target disambiguation was proposed in [112] where CNN with multi-exit branches was also used to identify source and target regions. This method was shown to be capable of learning special features focusing on the presence of interpolation artifacts and boundary inconsistencies. Figure 7 shows two examples of source-target disambiguation localization results generated by Wu et al.’s detector [111].

In [113] Liu et al. proposed one of the first copy-move detectors that used a CNN approach. Their proposal was partially based on conventional methods, by taking keypoints features such as Scale-Invariant Feature Transform (SIFT) or Speeded-Up Robust Features (SURF) as input for their network. One limitation was that this method had low performance when duplicated areas have a homogeneous content, because the keypoints could be hardly identified within such areas.

Very recently, Zhu et al. [114] proposed an adaptive attention and residual-based CNN to localize copy-move forgeries. The self-attention module allowed neurons to interact with each other to find out which neurons should receive more attention. Experiments showed comparable results with previous deep-learning approaches, but the problem of source-target disambiguation was not addressed.

Illumination direction, contrast and noise are usually inconsistent in splicing forgery, so the tampering traces could be found rather easily by the CNN. However, the source and target regions are derived from the same image in copy-move, accordingly the illumination and contrast would be highly consistent, which raises a greater challenge for copy-move detection based on CNN. This may be one reason for the fewer published papers focused on copy-move when compared with splicing.

The first part of Table 6 summarizes the existing deep-learning methods targeted at copy-move detection and localization. We show in the second-last column a comparison of localization performance of methods that reports results on both of the popular datasets of CoMoFoD [41] and CASIA [33]. Here CASIA means a specific subset of CASIA images with only copy-move falsification, which were properly selected and shared by the authors of the copy-move detection method of [111]. On CoMoFoD and CASIA datasets the comparison is fair with same set of images, evaluation protocol and metric. It is worth mentioning that the F1-score has become a commonly used evaluation metric of copy-move detection methods in part owing to Wu et al.’s paper [111], where the authors proposed a detailed evaluation protocol of copy-move localization performance with the F1-score as the metric. The proposed evaluation protocol and metric in [111] are later widely accepted and used by researchers in the community. From Table 6 we can see that methods of [111,114] have competitive results on both datasets of CoMoFoD and CASIA. Nevertheless, the method in [111] provides the additional capability of source-target disambiguation which may bring more information for the forensic analysis. Moreover, having a dedicated architecture for the copy-move forensic problem is helpful to achieve satisfying performance, in particular the block correlation module of [111] and the self-attention module of [114]. Finally, it can be observed that the performance is not high and there is still much room for improvement of copy-move localization results.

#### 4.2.3. Inpainting Detection

The inpainting technique can create plausible image forgeries which are difficult to spot by the naked eye. In contrast to copy-move where an image area is copied and pasted, in inpainting the falsified area is often filled with micro components (e.g., blocks of 7 by 7 pixels) extracted from different places of the image. These small blocks usually represent a kind of micro-texture and are combined in inpainting in a visually convincing way. Although the inpainting method can be used for inoffensive purposes such as repairing partially deteriorated images, it is used likewise in forgery creation, for instance for object removal to falsify an image or for erasing visible watermarks. Some splicing or copy-move detection algorithms could be exploited to detect inpainting forgeries, but in general they do not consider the particularity of inpainting and their performance remains not as good as expected.

To our knowledge the first method targeted at inpainting detection was proposed by Zhu et al. [117], where authors used an encoder–decoder network to predict the inpainting probability on each patch. Li and Huang [119] focused on detecting inpainting forgeries made by deep-learning methods (also known as deep-inpainting). Image high-pass residuals were fed to an FCN in which transpose convolutional layers were initialized with bilinear kernel.

Wang et al. [118] used a R-CNN, originally designed for object detection, to output a bounding box of the inpainted region along with a probability score. Very recently, the same authors [120] designed a multi-task CNN with two inputs, i.e., a Local Binary Pattern (LBP) image as the first input and the pixel values as the second one, for inpainting detection. This new network could produce a bounding box of inpainted area together with an estimated mask of forgery.

In [121] authors proposed an anomaly detection method by randomly removing partial image regions and reconstructing them with inpainting methods to detect a forgery. The authors used a U-Net-based encoder–decoder network to reconstruct the removed regions and output a tampering map in which each image is assigned an anomaly score according to the region with the poorest reconstruction quality. Meanwhile, Lu and Niu [122] published an object removal detection method by combining CNN and LSTM to detect inpainting with single and combined post-processing operations such as JPEG compression and Gaussian noise addition.

The second part of Table 6 provides a summary of the deep-learning-based forensic methods targeted at inpainting falsification. For experimental studies, the listed methods created an ad-hoc dataset from different databases of pristine images with different inpainting techniques and experimental protocols. This makes very difficult to carry out a fair comparison. We have made efforts and decided to report performances of compared methods under one typical experimental setting where in falsified images 10% of pixels were tampered with by inpainting falsification. We can see from the last column of Table 6 that methods in [117,119,120] achieved good inpainting localization performance. This may imply that the special inputs of high-pass residuals in [117,119] and of LBP features in [120] are effective in exposing traces left by inpainting techniques. We also observe that different methods tend to use different evaluation metrics, in part because authors of each method tested their method on an ad-hoc dataset created by themselves. This makes difficult to carry out easy and fair comparisons between different methods. The development of a high-quality open benchmarking dataset is desirable and will be beneficial for the advancement of the relevant research. Finally, it can be observed that localization performance is better for inpainting than copy-move (please compare the last two columns of Table 6). A possible reason is that in copy-move the falsified region is originally from the pristine part of the same image, while in inpainting the falsified region is a kind of new content created by inpainting algorithm though with attempt to mimic the pristine areas.

## 5. Other Specific Forensic Problems

This subsection is dedicated to the presentation about some other specific problems on which the image forensics research community has conducted extensive work. We divide them into three groups: (1) camera identification, (2) computer graphics image detection, and (3) detection of Deepfake images.

### 5.1. Camera Identification

**Figure 8:**
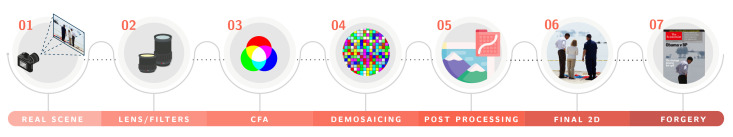
Illustration of typical pipeline of image acquisition and forgery creation.

A typical image acquiring process is shown in Figure 8. First, the light rays are redirected by the lens, then different filters such as anti-aliasing can be applied before the Color Filter Array (CFA) divides the light into one of the red (R), green (G) and blue (B) components per pixel. A demosaicing step is performed afterwards to reconstruct the full-color scene from the input samples taken by the previous step. Depending on the camera model and software, several post-processing operations such as white balancing, gamma correction and JPEG compression can take place. These post-processing steps contribute with important and distinctive clues to the image forensics field. When the final output image of camera is falsified to create a forgery, additional traces unique for each falsification are usually left behind.

The challenge of verifying the authenticity of an image can be tackled from different perspectives. One of them is approached by answering the following question: given an image, is it possible to find out the model of the camera with which the image was taken? Even though camera model, date and time, and other information can be found in the EXIF or in the JPEG header, in general it is not possible to consider such information as reliable and legitimate because image metadata can be easily modified. By contrast and as mentioned before, the traces of the post-processing steps carried out by each camera constitute important source of information that can be used to authenticate the image provenance in the image forensics field.

First deep-learning methods for camera identification were mainly dedicated to classifying patches produced by different cameras. Bondi et al. [123] used a CNN followed by an SVM to classify patches coming from different unknown cameras. In addition, with the output of their CNN they looked for anomalies in an image to search for forgeries. Tuama et al. [124] applied a high-pass filter in the first layer to suppress image content and obtain image residuals as input for a shallow CNN that was trained to learn to classify among different camera models. Due to the release of new camera models and the difficulty to keep an updated database, Bayar and Stamm [125] suggested an open-set scenario which aimed to predict an unseen camera device. The authors used a constrained initialization for the first layer of a CNN to infer whether the image was taken by an unknown device.

Ding et al. [126] proposed a multi-task CNN to predict information about brand, modes and devices from a patch. The authors used ResNet [88] blocks together with high-pass filter residuals as input for the network and with inputs of different sizes. In [127], authors used a shallow CNN for mobile camera identification in a multi-class challenging scenario. Experiments showed good forensic performance, but the performance diminished when devices came from a same manufacturer.

Methods in [128,129] both used Siamese network for this camera classification problem. There are multiple inputs in a Siamese network with the same architecture and same initial weights for each sub-network. Parameter updating is mirrored across all sub-networks. The purpose of this architecture is to learn the similarity of inputs. In [128], authors proposed a Siamese CNN to extract the camera unique fixed-pattern noise from an image’s Photo Response Non-Uniformity (PRNU) to classify camera devices and furthermore trace device fingerprints for image forgery detection. Sameer and Naska [129] worked on the scenario where annotated data (i.e., in this case image samples) were not available in big quantities and training had to be performed using a limited number of samples per class. This approach is called *few-shot* learning and refers to learning and understanding a new model based on a few examples. For this few-shot learning approach, a Siamese network was used to enhance classification accuracy of camera models. The intuition behind the Siamese network for this challenge is to form pairs of image patches coming from the same camera models to improve the training.

Table 7 gives a summary of the deep-learning-based camera identification techniques. In the table, we include the accuracy performance mainly on the Dresden dataset [17]. Even in cases where the same number of camera models was considered, the size of the patches was not the same, which makes difficult to deliver a fair comparison. Nevertheless, it must be mentioned that methods using smaller patch sizes such as 64×64 or smaller, combined with a bigger number of camera models present a bigger challenge due to the less information available on each patch and the bigger number of classes for the classification task. In addition, similar to the evaluation of image manipulation detection methods in Section 3.5, the experiments for evaluating camera model identification methods are usually conducted in a controlled laboratory setting with balanced classes. Therefore, in this case the simple accuracy metric has been widely used for the performance evaluation. It can be observed once again from Table 7 that special input features of high-pass residuals [124,125,126] and/or special first-layer design [125] appear to be effective in highlighting the subtle differences between the traces of different camera models, leading to a satisfying identification accuracy. Finally, we would like to mention our observations of two interesting trends regarding the research on camera model identification: (1) techniques such as few-shot learning would be helpful in realistic scenarios in which we have a limited number of annotated samples, and (2) the deep-learning methods are promising techniques to deliver a good camera model classification performance and may further help in the search of anomalies for image forgery detection.

### 5.2. Detection of Computer Graphics Images

**Figure 9:**
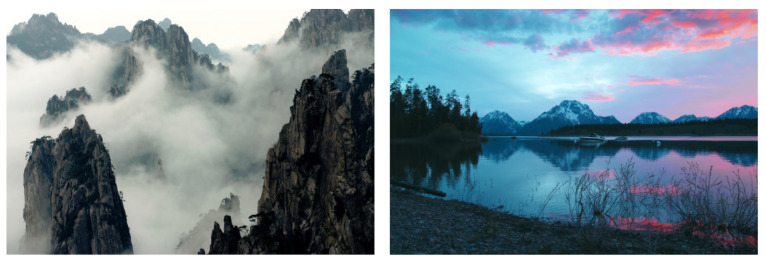
Examples to show the difficulty of visually differentiating between CGI (on the **left**) and an image taken by a camera (on the **right**). The CGI is from Tumblr forum (https://hyperrealcg.tumblr.com/post/112323738189/title-a-land-where-dreams-take-wings-artist accessed on 2 April 2021 ) and the camera image is from Reddit forum (https://www.reddit.com/r/EarthPorn/comments/4o9u03/no_filter_needed_grand_tetons_national_park_wy_oc/ accessed on 2 April 2021 ).

Computer graphics techniques produce visually plausible images of fictive scenes. Despite the benefits of CGI in virtual reality and 3D animation, it can also be used as false information thus affecting real-life decisions, and this situation is augmented with the fast dissemination of content enabled by the Internet. Consequently, the challenge of discerning between a real photograph and CGI has been explored by image forensics researchers. Figure 9 shows how challenging it is to distinguish between CGI and an image taken by a camera.

Rezende et al. [130] proposed a deep CNN taking advantage of transfer learning from ResNet-50 model to classify small patches of computer graphics images and real photographic images. Yu et al. [131] investigated for this CGI forensics problem the usage of a CNN without pooling layers. The authors of [57,132] proposed to use shallow CNNs in a patch-based manner. Rahmouni et al. [57] used a CNN with a customized pooling layer that computed statistics such as mean and variance followed by an SVM to detect CGI patches. In order to classify a whole image, a weighted voting strategy was applied to combine the local probabilities on patches of sliding windows to produce a final label. Quan et al. [132] proposed an end-to-end approach that used a Maximal Poisson-disk Sampling (MPS) method to crop patches in a lossless manner from a full-sized image. Nguyen et al. [133] continued with the sliding window approach to deal with high-resolution images using VGG-19 followed by multi-layer perceptron-based CNN as classifier. In [134], authors proposed an approach for discriminating CGI using high-pass residuals as input for a CNN.

He et al. [135] designed a two-input CNN-RNN taking the color and texture from YCbCr color space on each input to detect CGIs. In [136] authors investigated the usage of an Attention-Recurrent Neural Network (A-RNN) to classify CGIs in a local-to-global approach following the sliding window strategy and using the simple majority voting rule to produce a decision on a whole image. Nguyen et al. [137] studied the application of dynamic routing capsule networks [138] based on the VGG-19 model for detecting CGI. Capsule networks were able to identify objects that hold spatial relationship between features.

More recently, Zhang et al. [139] proposed a CNN containing a special block at input called hybrid correlation module composed of a 1×1 convolution layer followed by three blocks of convolutional layers, which would correlate channels and pixels in an attempt to detect CGIs. Meena and Tyagi [140] used the transfer learning approach from DenseNet-201 [141] followed by an SVM as classifier. In [142] authors made use of a shallow A-CNN with two inputs for CGI classification. Interestingly, the inputs for this network were pre-processed by a Gaussian low-pass filter as the authors wanted to focus on general patterns rather than local details. Quan et al. [143] designed a CNN combining SRM filters and Gaussian random weights as initializations for the first layer on a two-branch architecture. The authors also proposed to use the so-called negative samples created via gradient-based distortion to achieve a better generalization on test images created by unknown graphics rendering engines.

Table 8 summarizes the deep-learning-based CGI forensic techniques. In the last column of the table, we provide a performance comparison mainly for the Rahmouni dataset [57] and the He dataset [135]. The accuracy at patch level is used as the performance metric on these two datasets. This is mainly because similar to manipulation detection (Section 3.5) and camera model identification (Section 5.1), researchers consider a controlled experimental setting with balanced classes of natural image patches and CGI patches in the experiments; therefore in such cases the patch-level accuracy is a simple and appropriate metric. For the results in Table 8, the patch size is 60×60, 60×60 and 64×64 for method in [132,136,142], respectively. For other methods, results are reported for the only patch size listed in the column of “Input size”. In the case of the Rahmouni [57] dataset, we can see that in general accuracy improves as forensic method uses larger patches, with the two highest accuracy values achieved by [134,137] respectively on patches of 650×650 and 128×128. Regarding the performance on the He [135] dataset, we still have the same trend with better results achieved by [135,139] both on 96×96 patches, when compared to method [142] on 64×64 patches. In all, a larger patch size generally results in a better forensic performance of CGI detection but also leads to a higher computational cost. Future efforts could be devoted to the performance improvement on small patches and the aggregation strategy from patch-level results to image-level decision.

### 5.3. Deepfake Detection

**Figure 10:**
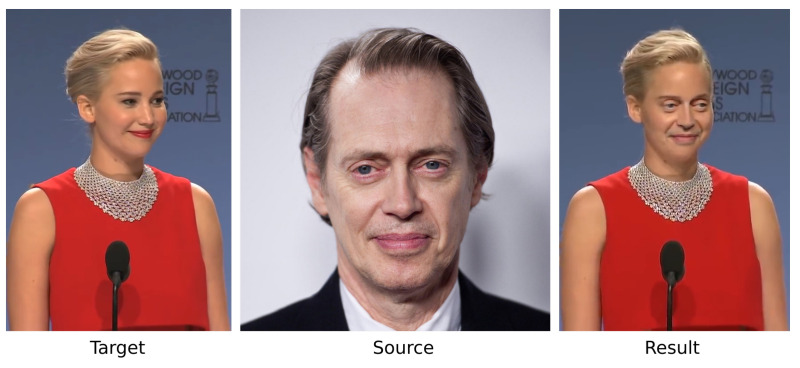
Example frame of a Deepfake video. The tool used to generate this video is available at the following webpage: https://faceswap.dev/ accessed on 2 April 2021, and the full resulting video can be viewed at https://www.youtube.com/watch?v=r1jng79a5xc accessed on 2 April 2021.

Lately, GAN models have been used in various applications and have transformed a time-consuming task previously reserved to high-skilled experts now to a simple and fast operation. One of such applications is to create synthetic yet visually realistic images and videos. GAN-generated multimedia contents are commonly known as *Deepfakes*, referring to the usage of a deep-learning model and the fabricated synthetic results. Majority of cases have been used to replace a person (or a person’s face) in an existing image or video with another person (or the face of this other person). Figure 10 illustrates the synthesis process realized by a GAN which replaces the face in the target (image on the left) by using a source (image in the middle) to generate the resulting frame (image on the right). Although benign material has been created for the illustrated example, this technique can have more serious impact in other situations, e.g., to create political distress. Recently a big amount of research activities has been dedicated to detecting GAN-generated fake content, mainly due to the easiness and impact of Deepfakes. In comparison with images, videos contain more information and different approaches have been proposed based on different kinds of clues for the detection of Deepfake videos.

First proposals in the literature [44,145,146,147] focused on the detection of GAN-generated images created by a specific GAN model. In [145], authors searched for statistical artifacts introduced by GAN with a pre-processing layer that extracted high-pass residuals. Marra et al. [146] tested the performance of some popular CNN-based image forensics methods for the detection of images created by GANs and shared in social networks. In [44], authors used a shallow CNN to detect Deepfakes and Face2Face [50] videos. Interestingly, Chan et al. [147] developed as first objective a GAN for video-to-video translation in dancing poses. Additionally, they developed a detector that would detect videos coming from their own model. In [148], authors compared several popular sophisticated architectures and a shallow CNN. Experiments showed that the shallow CNN had better performance in detecting Deepfakes.

Güera and Delp [149] proposed to use a CNN for frame feature extraction and an LSTM for temporal sequence analysis to detect Deepfake videos which contained inconsistent frames. Amerini et al. [150] investigated the use of optical flow vectors to detect discrepancies in motion across several frames using the PWC-Net model [151]. Optical flow is a vector computed on two consecutive frames to extract apparent motion between the observer and the scene itself. In a follow-up work [152], an LSTM was used in a sequence-based approach which exploited the dissimilarities between consecutive frames of Deepfake videos.

Other proposals like [53,153,154,155,156] focused on the spatial coherence and temporal consistency among different physiological features. In [153], authors designed a CNN to detect variations of heart rate extracted from face regions on different frames. Li et al.’s method [154] was based on the observation that faces in Deepfake videos had a lower rate of blinking in comparison with real videos. This occurred in early GAN-generated videos for which the GAN was trained on faces with open eyes. The authors carried out a couple of pre-processing steps to locate the eyes and used this feature as input for an LSTM to detect a lower or higher rate of blinking as a telltale of Deepfake videos. Korshunov et al. [53] proposed an LSTM to search for anomalies between the audio and mouth movements. The method in [155] went in the same direction by comparing mouth shapes with the sound associated with M, B an P phonemes which required complete mouth closure and were in many cases incorrectly synthesized. Recently, Mittal et al. [156] went a step forward using a Siamese network to look for anomalies between the audio and video and combined it with the affective cues of both inputs to learn the differences between real and Deepfake videos.

The signal-level artifacts introduced during the synthesis were investigated for the detection of fake content. Li et al. [157] focused on artifacts at face boundaries by exploiting the fact that most existing face tampering methods shared a common blending operation. Meanwhile in [158], authors exploited the inconsistencies between warped face area and the surrounding background. The method in [159] adopted Gaussian noise extraction as a pre-processing step for a CNN, enforcing the network to learn more meaningful features about GAN traces.

In [160] a multi-task CNN was proposed to detect fake faces and to segment tampered-with areas. Dang et al. [161] investigated the use of attention mechanism for the detection and segmentation of tampered-with faces. In [162], authors used deep transfer learning for face swapping detection. Hsu et al. [163] made use of a so-called Common Fake Feature Network (CFFN) consisting of several dense units and a Siamese network for Deepfake detection. One limitation was that the CFFN may fail when the fake features of the results of a new GAN were significantly different from most of those used in the training phase.

To overcome data scarcity, refs. [164,165] proposed some solutions. Fernandes et al. [164] used a Attribution Based Confidence (ABC) metric to detect Deepfake videos with a deep model only trained on original videos. Khalid and Woo [165] formulated the challenge as a one-class anomaly detection problem by using a Variational Autoencoder trained only on real face images and subsequently detected Deepfakes as anomalies.

More recently, Wang et al. [166] used the well-known ResNet50 with careful data preparation to study the artifacts left by GANs. Their method demonstrated good generalization performance on unseen Deepfake content. In [167], authors designed a two-branch CNN to exploit the distribution differences between pixels in the face region and the background. Masi et al. [168] proposed a two-branch LSTM to combine color and frequency information. A multi-scale Laplacian-of-Gaussian operator was used in their method, which acted as a band-pass filter to amplify the artifacts.

Table 9 provides a summary of Deepfake detection methods presented above. In particular, in the table we present the main cue used by each method, by grouping cues into several categories as spatial context, generator traces, physiology-inspired, inter-frame consistency, and anomaly classification. We show performance comparison mainly on the two most common datasets used among all methods, i.e., FaceForensics [50] and FaceForensics++ [51]. The simple accuracy metric is the most commonly used evaluation metric for Deepfake detection methods, still because researchers mainly consider a controlled experimental setting with balanced classes of real and fake samples. Other metrics, e.g., mAP and AUC, have also been used for instances by researchers originally coming from the computer vision field. Even though accuracy is the most common metric, different settings were used for each method. Specifically, video compression levels were not the same for methods that conducted tests on a same dataset. H.264 compression was sometimes applied on the testing set providing different subsets with different compression levels. Additionally, lossless compression was used in some cases. Given the fact that Deepfake traces can get lost after lossy compression, uncompressed settings may present better results than scenarios with compression. Finally, compression level was not specified in all methods. Therefore, a direct and fair comparison is difficult. Nevertheless, we discuss some interesting points on these methods. We can see that most methods achieved a good performance on a binary classification for GAN-generated images. In the case of videos as input (cf., column of “Video”) and using FaceForensics++ as dataset, the use of an architecture that can track changes among frames, such as LSTM in the method of [168], leads to very good performance. On images (cf., column of “Image”), results from [166] show that traces of current GANs are easy to detect. In addition, with data augmentation techniques detectors can achieve a good generalization on unseen data created by unknown Deepfake generation tools. A final remark is that the different metrics, e.g., accuracy, AUC and mAP, are not directly comparable. First, there is a clear difference between the threshold-dependent accuracy metric and the more comprehensive AUC and mAP metrics which in theory consider all possible threshold values. Second, although under certain conditions and with additional information of the classification system AUC and mAP can have an approximate relationship [169], in general these two metrics are not easily convertible to each other. This highlights the importance of open-source policy of forensic methods and free availability of high-quality datasets. With open implementations and datasets, it will be possible to carry out reliable evaluation of existing and future methods even on new datasets and with new metrics.

## 6. Anti-Forensics

Anti-forensics also called counter-forensics aims at defeating the analysis and examination of forensic methods. Different techniques can be adopted by a smart and determined adversary to modify an image while attempting to prevent image forensics tools from getting useful clues on manipulations, falsifications, source devices, etc.

Early research [171,172] showed that CNN models are vulnerable to adversarial attacks. In the deep-learning-based anti-forensics field this has been translated to the use of GANs to recreate or hide different cues with visually imperceptible distortions.

Güera et al. [173] proposed a method to slightly modify images to alter their estimated camera model when analyzed by a CNN. The authors showed that adversarial-attack-based approaches such as Jacobian-based Saliency Map Attack (JSMA) and Fast Gradient Sign Method (FGSM) are capable of misleading CNN models that have been trained to perform camera model identification.

In [174], Chen et al. proposed a white-box scenario where information on the forensic tool and camera model is known. A GAN was proposed to modify traces used to identify a camera model. Additionally, they introduced a new loss function focused on both fooling a CNN-based detector of camera models and introducing minimum distortion into the image. Later, the same authors proposed in [175] the usage of GAN for two scenarios: a data-dependent scenario where camera model is known and a data-independent one where no information is available. In both cases a generative model was used to fool CNN-based camera model identification methods.

An anti-forensic method for recaptured image detection was proposed by Zhao et al. [176]. The authors proposed to employ Cycle-GANs typically used for image translation to accomplish this anti-forensic task of hiding traces of image recapturing. In their work, high-pass filters were used within the model to improve the anti-forensic performance. Moreover, the loss function was also adapted to ensure that the image content would not be changed too drastically.

Other proposals focused on concealing the traces left by routine image manipulations. Kim et al. [177] proposed a GAN model which was able to reproduce and hide the cues left by median filtering operation. Meanwhile, Wu and Sun [178] investigated the use of GANs and a tuned loss function to hide the traces left by multiple image manipulation operations. Uddin et al. [179] proposed a GAN-base anti-forensic method against double JPEG compression detection. Results showed that detection accuracy could be reduced from 98% to 85% by using the proposed method.

In [180], authors designed a small GAN architecture to prevent CGIs from being correctly detected. In this approach, the first layer of the discriminator was initialized with 2 *Sobel* filters to guide the network to concentrate more on the texture information of the input image.

Barni et al. [181] presented an analysis on the transferability of anti-forensic attacks. Their results showed that in most cases, attacks are not transferable, which facilitates the design of appropriate counter measures against anti-forensics. This is particularly true when an anti-forensic adversary does not have full knowledge of the targeted forensic method.

Table 10 provides a summary of deep-learning-based anti-forensic methods presented above. In particular, in the table we present the main component used by each method and the targeted forensic problem. We can see that the current trend is to design GAN-based anti-forensic algorithms against camera model identification methods and detectors of routine image manipulations. We expect to see in the near future interesting anti-forensic works considering more advanced tampering operations.

## 7. Concluding Remarks

Through this review, we provide a general understanding of the detection methods in the image forensics field. We collected and presented many deep-learning-based methods divided into three broad categories, with a focus on the different characteristics that are particular for the image forensics approaches. It can be observed that a pre-processing step to obtain a certain feature or a special initialization on the network’s first layer have been used in many pioneer works and still exist in recent ones. It is interesting to see that these characteristics are mainly present in the manipulation, falsification, camera identification and CGI detection methods but scarcely seen in the Deepfake detection works. We have not found clear reasons to explain this observation, and it would be interesting to carry out theoretical studies and practical comparisons with and without the use of pre-processing step and with different initializations of the first layer, for various forensic problems. This is a research opportunity to be explored in our future work. As any arms race scenario where two opponents, in this case a forger and a forensic investigator, try to make their respective actions successful, both sides will keep evolving with new technologies and challenges. Deep learning has brought a tremendous advance due to its ability to automatically learn useful features from available data and this strength has been used on both sides and their competition will be continued in the future.

One promising working direction is that it is beneficial to gain access to real-life forgery datasets that include ground-truth masks with a vast number of samples. Currently, depending on the forensic problem we want to study, existing datasets may have a limited number of examples or focus on a small range of devices or subjects. Although data scarcity has been tackled with the few-shot learning approach, the generalization problem may still be in game. In the case of Deepfake detection, a very popular research topic as we see from the large number of recent works collected in this review, high-quality datasets are becoming more and more available because of the involvement and commitment of big companies.

We also believe that although single works can obtain good performance, the combination of several domains or features will be of huge importance in the future. We have listed some works that combine the usage of image and audio features to detect Deepfakes, and probably these works would benefit from other features or strategies if properly combined. To this end, the availability of open-source implementation of existing methods is of paramount importance.

Another interesting future research topic is the development of different counter-forensic methods which we believe have a right of existence. Indeed, the creation of tools to deceive forensic detectors adds another interesting and important player in the game who challenges the detectors of fake multimedia content. As we saw in Section 6, almost all existing deep-learning-based anti-forensic methods make use of a GAN model which has proved to show good results. Nevertheless, different strategies could be explored to realize the objective of removing forensic cues, from the design of appropriate network architectures to the explicit analysis and removal of forensic traces with customized layers and loss function. Additionally, it is interesting to notice that special initializations on the first layer of a network architecture have also been used in the anti-forensics field. The resilience of forensic detectors would be improved by considering the attacks of anti-forensic methods. We believe that the competition between the two sides of forensics and anti-forensics would be beneficial for the advancement of both subjects and is an interesting topic to follow.

In all, we think that the image forensics research presents big challenges and opportunities for the future in which we hope to see more deep-learning-based methods to take better account of the particularities of the image forensics field.

## Figures and Tables

**Table 1:** Summary of datasets of original image data and falsified image data. In the “Format” column we show the compression type of images by using the first character, with “U” for uncompressed, “C” for lossless compression, and “L” for lossy compression. “Grayscale/color and bit depth” is color coded as follows: 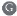 grayscale, 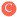 color, followed by the number of bits of the grayscale or color information. “GT” stands for “Ground-truth”. The “Content ratio” column shows the number of pristine/tampered-with images.

Type	Name	Size	Format	Grayscale/Color and Bit Depth	Content Ratio	Operations				GT Mask
						Copy-Move	Splicing	Inpainting	Others	
Original data	BossBase [16]	512×512	U-PGM	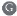 8-bit	10K					N/A
	UCID [15]	512×384, 384×512	U-TIFF	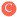 24-bit	1338					N/A
	Landsat [23]	650×650; 5312×2988	C-TIFF	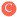 48-bit	Ongoing					N/A
	MIT SUN [28]	200×200	L-JPEG	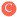 24-bit	130,519					N/A
	NRCS [42]	1500×2100	U-TIFF, L-JPEG	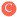 24-bit 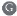 8-bit	11,036					N/A
	MS COCO [25]	Various	L-JPEG	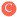 24-bit	328K					N/A
	CelebA [29]	64×64; 512×512	L-JPEG	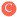 24-bit	200K					N/A
	CelebAHQ [30]	512×512	L-JPEG	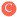 24-bit	30K					N/A
	RAISE [18]	4288×2848	C-TIFF, U-NEF	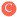 36-bit	8156					N/A
	Dresden [17]	3039×2014; 3900×2616	L-JPEG, U-NEF	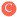 24-bit, 36-bit	14K					N/A
	MICHE-I [19]	640×480; 2322×4128	L-JPEG	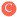 24-bit	3732					N/A
	Kaggle Camera [20]	1520×2688; 4160×3120	L-JPEG, C-TIFF	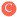 24-bit	2750					N/A
	Vision [21]	960×720; 5248×3696	L-JPEG	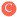 24-bit	34427					N/A
Falsified data	Columbia gray [31]	128×128	U-BMP	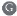 8-bit	1845/912		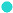			No
	IEEE IFS-TC [34]	1024×768; 3000×2500	C-PNG	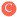 24-bit	1050/1150	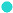	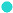			Yes
	CASIA v1 [33]	384×256	L-JPEG	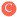 24-bit	1725/925		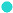			No
	CASIA v2 [33]	240×160; 900×600	L-JPEG, U-BMP, U-TIFF	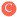 24-bit	7491/5123	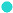	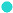			No
	NIST Nimble 16 [36]	500×500; 5616×3744	L-JPEG	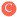 24-bit	560/564	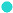	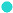	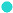		No
	NIST Nimble 17 [37]	60×120; 8000×5320	U-NEF, C-PNG, U-BMP, L-JPEG, U-TIFF	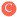 36-bit, 24-bit	2667/1410	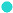	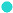	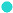		No
	Coverage [40]	400×486	C-TIFF	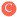 24-bit	100/100	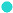				Yes
	Columbia color [32]	757×568; 1152×768	U-TIFF	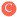 24-bit	183/180		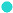			Yes
	Carvalho [35]	2048×1536	C-PNG	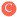 24-bit	100/100		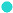			Yes
	Realistic (Korus) [38]	1920×1080	C-TIFF	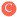 24-bit	220/220	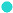	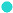			Yes
	CoMoFoD [41]	512×512; 3000×2000	C-PNG	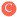 24-bit	260/260	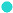				Yes
	VIPP [39]	300×300; 3456×5184	U-TIFF	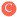 24-bit	68/69		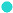		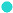	Yes

**Table 2:** Datasets of artificially generated data.

Type	Name	Size	Format	Codec	Content Ratio	Media	
						Video	Image
CGI	Columbia [43]	700×500; 3000×2000	JPEG	-	1600/1600		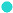
	Rahmouni [57]	1920×1080; 4900×3200	JPEG	-	1800/1800		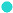
CGI and Deepfakes	Faceforensics [50]	480p	MP4	H.264	1000/1000	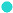	
Deepfakes	UADFV [49]	294×500	MP4	H.264	49/49	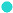	
	Faceforensics++ [51]	480p,720p, 1080p	MP4	H.264	1000/4000	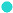	
	Afchar [44]	854×480	JPEG	-	7250/5100		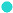
	PGGAN [30]	64×64; 1024×1024	JPEG	-	-/100 K		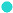
	Deepfake TIMIT [53]	64×64; 128×128	AVI	H.264	-/620	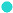	
	CelebDF [52]	Various	MP4	H.264	509/5639	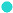	
	DFDC [55]	180p; 2160p	MP4	H.264	1131/4113	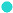	
	DFD [56]	1080p	MP4	H.264	363/3068	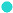	

**Table 3:** Image manipulation detection methods. Network depth describes the number of convolutional blocks with C for a convolutional layer, or M for mlpconv layer, followed by an activation function and pooling layer, as well as the number of fully connected blocks denoted by an F (fully connected layer and activation function). MF stands for median filtering, DJPEG for double JPEG, CE for contrast enhancement, GIPO for general-purpose image processing operations, and approach is color coded as: 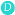 detection, 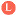 localization. Dataset is color coded as follows: 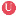 UCID [15], 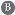 BOSSBase [16], 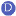 Dresden [17], 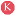 RAISE [18], 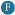 MS COCO [25], 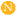 NRCS [42], and 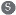 when it is an ad-hoc dataset created by authors of the original paper. In the column of “Patch performance”, Acc. stands for accuracy, TPR stands for true positive rate, and AUC stands for area under the curve (all in %).

Problem	Method	Network Depth	Input Feature	Special CNN Design	Input Size	Approach	Dataset	Patch Performance
MF	[58]	5C-2F	MFR	N/A	64×64, 32×32	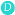	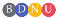	Acc. 85.14
	[59]	2M-3C	Upscaled values	mlpconv	64×64, 32×32	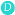	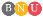	Acc. 89.96
DJPEG	[63]	4C-3F	DCT features	N/A	128×128	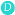	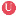	Acc. 99.48
	[62]	2C-2F	DCT features	Customized 3 × 1 kernels	64×64, 128×128, *…*, 1024×1024	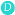	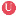	AUC 100.00
	[64]	3C-2F	Noise residuals or DCT features	N/A	64×64, 256×256	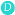	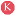	Acc. 96.30
	[65]	2C-2F, 3C-1F	DCT features, pixel values	Two-branch CNN	64×64	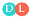	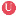	Acc. 99.60
	[66]	4C-3F, 3F	DCT features, quantization tables	Two-branch CNN	256×256	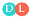	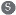	Acc. 92.76
CE	[68]	9C-1F	Pixel values	N/A	64×64	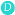	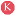	AUC 99.7
	[69]	3C-2F	GLCM	N/A	256×256	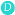	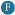	TPR 99.80
	[70]	4C-2F	GLCM	N/A	256×256	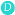	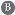	AUC 99.40
GIPO	[71]	5C-2F	Pixel values	Constrained 1st layer	256×256, 64×64	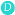	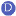	Acc. 94.19
	[72]	5C-2F	Pixel values	Special init. for 1st layer	64×64	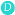	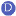	Acc. 93.71
	[74]	5C-2F, 6C	Pixel values	Scaling for 1st layer	64×64	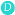	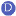	Acc. 96.02

**Table 4:** Multipurpose image falsification detection methods. Loc. level describes whether the localization is performed in a pixel-, block- or bounding-box-wise manner. Dataset is color coded as follows: 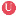 UCID [15], 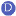 Dresden [17], 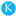 Kaggle Camera Challenge [20], 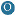 Vision [21], 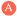 Landsat on AWS [23], 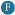 MS COCO [25], 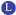 Columbia gray [31], 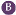 Columbia color [32], 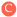 CASIA [33], 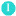 IEEE Forensics Challenge [34], 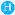 Carvalho [35], 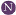 NIST 16 [36], 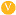 Coverage [40], and 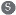 when it is an ad-hoc dataset created by authors of the original paper. In the last two columns of performance (Perf.) on respectively CASIA [33] dataset and NIST 16 [36] dataset, F1 stands for F-1 score, Acc. stands for accuracy, and AUC stands for area under the curve (all in %).

Method	Input Feature	Init. First Layer	Input Size	Loc. Level	Dataset	Network Type	Perf. on CASIA	Perf. on NIST 16
[78]	Pixel values	SRM filters	128×128	pixel	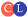	CNN - SVM	Acc. 97.8	-
[75]	Wavelet features	Random init.	32×32	block	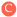	Autoencoder	Acc. 91.1	-
[79]	Steganalysis features	Random init.	128×128	pixel	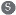	CNN - SVM	-	-
[83]	Pixel values	Random init.	64×64	block	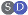	CNN	-	-
[80]	Radon features	Random init.	64×64, 128×128	pixel	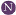	LSTM	-	Acc. 94.9
[92]	Resampling features	Random init.	64×64	pixel	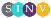	CNN	-	Acc. 89.4
[86]	Pixel values, noise features	Random init.	224×224	bbox	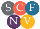	Multi-branch	AUC 79.5	AUC 93.7
[85]	Pixel values	Random init.	64×64	block	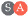	GAN-SVM	-	-
[84]	Pixel values, Noiseprints	Random init.	44×44, 64×64	pixel	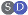	CNN	-	-
[81]	Resampling features	Random init.	Resized 256×256	pixel	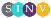	LSTM	-	Acc. 94.8
[87]	Pixel values	SRM filters, Bayar filters, Random init.	256×256, 512×512	pixel	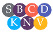	Multi-branch	AUC 81.7	AUC 79.5
[89]	Pixel values, Noiseprints	Random init.	960×720; 4640×3480	pixel	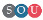	CNN incremental learning	-	-
[91]	Pixel values	Random init.	224×224	pixel	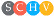	GAN-CNN	F1 57.4	-

**Table 5:** Targeted splicing detection methods. AE stands for autoencoder. Dataset is color coded as follows: 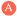 Landsat on AWS [23], 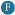 MS COCO [25], 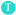 SUN 2012 [28], 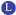 Columbia gray [31], 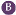 Columbia color [32], 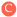 CASIA [33], 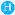 Carvalho [35], 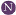 NIST 16 [36], 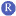 Realistic (Korus) [38], 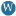 On-the-wild websites, and 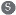 when it is an ad-hoc dataset created by authors of the method (information of source images used for dataset creation may be provided in the original paper). In the last two columns of performance (Perf.) on respectively Carvalho [35] and CASIA [33], F1 stands for F-1 score, mAP stands for mean average precision, and Acc. stands for accuracy (all in %).

Method	Input Feature	Input Size	Dataset	Network Type	Backbone Architecture	Perf. on Carvalho	Perf. on CASIA
[93]	SRM features	768×1024	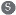	AE	Own	-	-
[95]	Pixel values	256×256	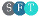	CNN	VGG-16	-	-
[94]	SRM features	720×1280	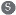	AE-LSTM	Own	-	-
[97]	Pixel values	224×224	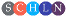	FCN	VGG-16	F1 47.9	F1 54.1
[100]	EXIF metadata, pixel values	128×128	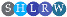	CNN	ResNet-v2	mAP 51.0	-
[101]	Illuminant maps	224×224	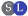	CNN-SVM	ResNet-v1	-	-
[98]	Pixel values	224×224	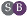	FCN	VGG-16	-	-
[103]	Pixel values	384×384	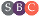	CNN (U-Net)	ResNet-v1	-	F1 84.1
[102]	Pixel values	512×512	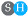	GAN (U-Net)	VGG-16	mAP 48.0	mAP 74.0
[106]	Pixel values	256×256	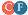	GAN	VGG-16	-	F1 90.8
[105]	Pixel values	70×70	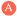	GAN	Pix2Pix	-	-
[107]	SRM features for 1st layer init.	128×128	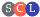	CNN-SVM	Own	-	Acc. 97.0

**Table 6:** Targeted detectors of copy-move and inpainting falsifications. S-T disam. means source-target disambiguation. Dataset is color coded as follows: 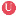 UCID [15], 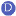 Dresden [17], 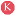 RAISE [18], 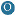 Vision [21], 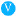 MvTec [22], 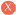 Oxford [24], 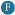 MS COCO [25], 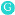 ImageNet [26], 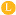 MIT Place [27], 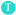 SUN 2012 [28], 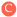 CASIA [33], 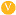 Coverage [40], 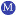 CoMoFoD [41], 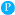 ROME patches [115], 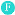 CMFD [116], and 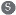 when it is an ad-hoc dataset. We show in the second-last column performances of F-1 scores (in %) for copy-move detectors on respectively CoMoFoD [41] and CASIA [33] datasets with the following format: F1CoMoFoD / F1CASIA. For inpainting forensic methods, we show in the last column the localization performance (mean average precision (mAP), F-1 score (F1), area under the curve (AUC) or accuracy (Acc.), all in %) for one typical setting of inpainted images with 10% of pixels tampered with by inpainting.

Method	Input Features	Input Size	Localization Level	Dataset	Backbone Architecture	Performance Copy-Move	Performance Inpainting
**Copy-move**							
[108]	Pixel values	256×256	Detection	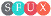	AlexNet	-	N.A.
[110]	Pixel values	256×256	Image	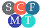	VGG-16	31.3 / 14.6	N.A.
[113]	Keypoints	51×51	Pixel	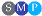	Own	-	N.A.
[111]	Pixel values	256×256	Pixel, S-T disam.	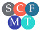	VGG-16	49.3 / 45.6	N.A.
[112]	Pixel values	64×64	Pixel, S-T disam.	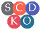	ResNet-V1	-	N.A.
[114]	Pixel values	256×256	Pixel	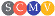	VGG-16	50.1 / 45.5	N.A.
**Inpainting**							
[117]	High-pass residuals	256×256	Pixel	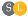	Own	N.A.	mAP 97.8
[118]	Pixel values	128×128	bbox	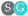	ResNet-V1	N.A.	F1 91.5
[119]	High-pass residuals	Various	Pixel	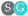	ResNet-V1	N.A.	F1 97.3
[120]	LBP, pixel values	256×256	Pixel & bbox	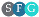	Own	N.A.	mAP 97.8
[121]	Pixel values	256×256	Pixel	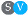	Own	N.A.	AUC 94.2
[122]	Pixel values	256×256	Pixel & bbox	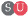	Own	N.A.	Acc. 93.6

**Table 7:** Camera identification methods. ET means extremely randomized trees. Dataset is color coded as follows: 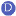 Dresden [17], 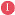 MICHE-I [19], 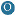 Vision [21], and 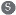 when it is an ad-hoc dataset. In the last column we show the accuracy performance on the Dresden dataset [17], except for two methods for which the dataset icon is given in the corresponding rows in the last column. We provide information about the number of tested camera models and the accuracy (in %) with the format Number of Camera Models: Accuracy.

Method	Input Features	Initialization	Input Size	Dataset	Network Type	Performance
[124]	High-pass residuals	Random init.	256×256	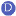	CNN	12: 98.0
[123]	Pixel values	Random init.	64×64	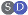	CNN-SVM	18: 93.0
[125]	High-pass residuals	Bayar’s constrained	256×256	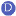	CNN-SVM CNN-ET	10: 93.9
[127]	Pixel values	Random init.	32×32	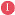	CNN-SVM	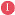 3: 91.1
[128]	Pixel values	Random init.	48×48	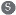	Siamese	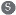 3: 100.0
[126]	High-pass residuals	Random init.	48×48	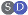	Multi-scale CNN	14: 97.1
[129]	Pixel values	Random init.	64×64	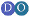	Siamese	10: 87.3

**Table 8:** CGI detection methods. Dataset is color coded as follows: 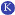 RAISE [18], 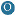 Vision [21], 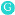 ImageNet [26], 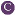 Columbia CGI [43], 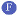 MesoNet [44], 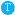 Artlantis [45], 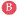 Corona [46], 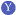 VRay [47], 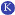 Autodesk [48], 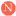 FaceForensics [50], 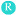 Rahmouni [57], 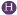 He [135], 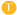 Tokuda [144], 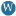 Web images, and 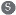 when it is an ad-hoc dataset. In the last column we show the performance in terms of patch-level accuracy (in %), except for method [143] for which HTER (half total error rate, in %) is used, on the Rahmouni [57] dataset 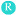, the He [135] dataset 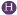, and ad-hoc dataset 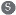 constructed or considered by authors of the corresponding method. In many cases the ad-hoc dataset 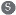 is a customized combination of the image sets listed in the third column of “Dataset”.

Method	Input Size	Dataset	Network Type	Backbone Architecture	Performance
[130]	224×224	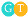	CNN-SVM	ResNet-50	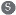 Acc. 94.1
[131]	32×32	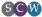	CNN	VGG-16	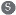 Acc. 98.0
[57]	100×100	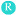	CNN-SVM	Own	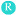 Acc. 84.8
[132]	30×30, *…*, 240×240	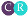	CNN	Own	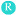 Acc. 94.8
[134]	650×650	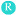	CNN	Own	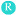 Acc. 99.9
[133]	100×100	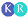	Two-input CNN-RNN	VGG-19	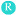 Acc. 96.5
[135]	96×96	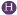 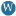	CNN-RNN	ResNet-50	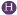 Acc. 93.9
[136]	30×30,..., 240×240	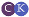	A-RNN	Own	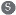 Acc. 94.9
[137]	128×128	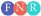	Capsule	VGG-19	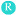 Acc. 97.0
[139]	96×96	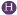	CNN	Own	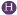 Acc. 94.2
[140]	224×224	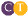	CNN	DenseNet-201	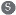 Acc. 94.1
[142]	32×32, 64×64	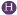	Two-input A-CNN	Inception	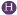 Acc. 87.8
[143]	233×233	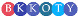	Two-branch CNN	Own	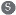 HTER 1.31

**Table 9:** Deepfake detection methods. Dataset is color coded as follows: 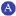 CelebA [29], 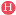 CelebAHQ [30], 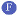 MesoNet [44], 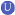 UADFV [49], 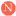 FaceForensics [50], 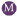 FaceForensics++ [51], 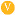 CelebDF [52], 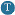 DeepfakeTIMIT [53], 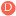 DFDC [55], 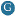 DFD [56], 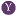 CycleGAN [170], and 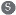 when it is an ad-hoc dataset. We show in the last column performance mainly on FaceForensics [50] dataset 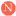 and FaceForensics++ [51] dataset 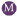, as well as on other datasets considered or constructed by authors of the corresponding method. In some cases, ad-hoc dataset 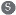 used for performance evaluation comprises fake samples generated by authors of the corresponding method with existing Deepfake generation tools. Acc. stands for accuracy, AUC for area under the curve, EER for equal error rate, TPR for true positive rate, Prec. for precision, and AP for average precision (all in %).

Method	Input Size	Dataset	Network Type	Backbone Architecture	Image	Video	Cue					Performance
							Spatial	GAN Trace	Physiology	Inter-Frame	Anomaly	
[145]	256×256	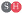	CNN	Own	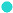		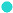					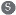 Acc. 99.4
[146]	256×256	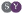	CNN	XceptionNet	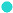			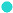				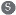 Acc. 94.5
[154]	224×224	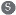	CNN-LSTM	VGG16		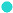			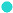			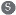 AUC 99.0
[149]	299×299	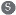	CNN-LSTM	Inception V3		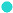				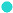		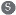 Acc. 97.1
[44]	256×256	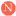	CNN	Inception		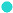		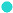				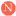 Acc. 95.3
[148]	1024×1024	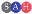	CNN	VGG16, ResNet110, etc.	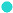			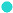				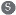 AUC 94.0
[158]	224×224	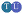	CNN	VGG16, ResNet50,101	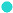	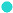	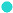					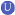 AUC 97.4
[147]	256×256	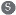	CNN	Own		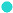		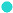				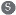 Acc. 97.0
[150]	224×224	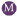	CNN	PWC-Net		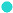				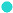		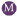 Acc. 81.6
[153]	128×128	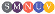	CNN	Own		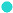			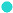			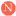 Acc. 82.5 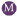 Acc. 80.6
[53]	720×576, 512×384	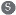	CNN-LSTM	Own		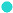			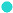			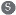 ERR 9.8
[160]	256×256	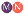	AE-CNN	Own	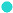	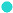	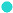					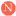 Acc. 90.3 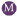 Acc. 84.9
[159]	128×128	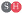	CNN	Own	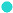	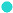		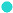				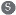 Acc. 95.5
[162]	224×224	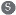	CNN	ResNet18	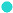	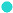	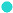					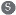 Acc. 99.9
[155]	128×128	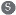	CNN	XceptionNet		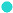			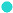			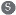 TPR 97.8
[157]	64×64	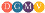	CNN	XceptionNet	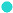	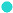		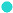				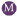 AUC 98.5
[161]	299×299	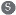	CNN	XceptionNet, VGG16	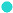	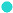	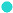					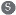 AUC 99.7
[152]	256×256	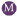	LSTM	Inception V3		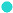				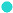		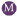 Acc. 94.3
[164]	224×224	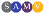	ABC-CNN	ResNet50	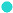			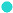				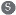 Acc. 96.0
[163]	64×64	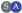	Siamese-CNN	Own	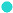			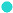				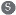 Prec. 98.8
[165]	100×100	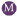	VAE	One-Class VAE	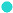						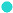	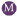 Acc. 98.2
[167]	224×224	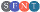	CNN	ResNet18	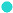		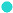					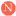 Acc. 99.4
[168]	224×224	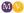	LSTM	Own		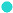	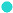					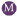 Acc. 96.4
[156]	Unknown	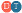	CNN	Own		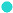			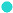			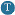 AUC 96.3
[166]	224×224	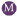	CNN	ResNet50	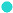		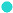					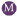 AP 98.2

**Table 10:** Anti-forensic methods. The column of “Backbone strategy” shows the main technical component used in each method. Dataset is color coded as follows: 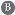 BOSSBase [16], 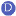 Dresden [17], 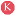 RAISE [18], 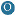 Vision [21], 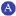 CelebA [29], 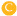 Cao [182], 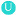 Agustsson [183], and 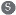 when it is an ad-hoc dataset created by authors of the original paper.

Method	Problem	Backbone Strategy	Input Size	Dataset
[173]	Camera identification	JSMA, FGSM	32×32	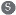
[174]		GAN	256×256	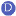
[175]		GAN	64×64, 227×227	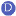
[176]	Recaptured image detection	Cycle-GAN	256×256	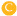
[177]	Median filtering	GAN	64×64, 227×227	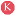
[178]	Multiple image manipulations	GAN	256×256	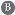
[179]	Double JPEG compression	GAN	512×384	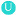
[180]	CGI detection	GAN	178×218	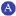
[181]	Attack transferability	GAN	128×128	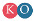

## Data Availability

Not applicable.
